# A Computational Model for the Cold Response Pathway in Plants

**DOI:** 10.3389/fphys.2020.591073

**Published:** 2020-11-05

**Authors:** Ruqiang Zhang, Didier Gonze, Xilin Hou, Xiong You, Albert Goldbeter

**Affiliations:** ^1^College of Horticulture, Nanjing Agricultural University, Nanjing, China; ^2^Unité de Chronobiologie Théorique, Faculté des Sciences, Université Libre de Bruxelles (ULB), Brussels, Belgium; ^3^College of Sciences, Nanjing Agricultural University, Nanjing, China

**Keywords:** cold response pathway, cold tolerance, C-repeat-binding factor, computational model, systems biology, plant cold acclimation

## Abstract

Understanding the mechanism by which plants respond to cold stress and strengthen their tolerance to low temperatures is an important and challenging task in plant sciences. Experiments have established that the first step in the perception and transduction of the cold stress signal consists of a transient influx of Ca^2+^. This Ca^2+^ influx triggers the activation of a cascade of phosphorylation-dephosphorylation reactions that eventually affects the expression of C-repeat-binding factors (CBFs, notably CBF3), which were shown in many plants to control resistance to cold stress by regulating the expression of cold-regulated (COR) genes. Based on experimental observations mostly made on *Arabidopsis thaliana*, we build a computational model for the cold response pathway in plants, from the transduction of the cold signal *via* the transient influx of Ca^2+^ to the activation of the phosphorylation cascade leading to CBF3 expression. We explore the dynamics of this regulatory network by means of numerical simulations and compare the results with experimental observations on the dynamics of the cold response, both for the wild type and for mutants. The simulations show how, in response to cold stress, a brief Ca^2+^ influx, which is over in minutes, is transduced along the successive steps of the network to trigger the expression of cold response genes such as CBF3 within hours. Sometimes, instead of a single Ca^2+^ spike the decrease in temperature brings about a train of high-frequency Ca^2+^ oscillations. The model is applied to both types of Ca^2+^ signaling. We determine the dynamics of the network in response to a series of identical cold stresses, to account for the observation of desensitization and resensitization. The analysis of the model predicts the possibility of an oscillatory expression of CBF3 originating from the negative feedback exerted by ZAT12, a factor itself controlled by CBF3. Finally, we extend the model to incorporate the circadian control of CBF3 expression, to account for the gating of the response to cold stress by the plant circadian clock.

## Introduction

Low temperature has adverse effects on the survival, growth, and development of plants (Chew and Halliday, 2010). In order to survive exposition to low temperature, plants have evolved sophisticated mechanisms to sense the seasonal, daily, and rapid fluctuations in temperature, and to adjust their physiology appropriately (Kaplan et al., 2004; Chinnusamy et al., 2006). In many temperate plants, cold acclimation increases freezing tolerance after exposure to nonfreezing low temperatures (Thomashow, 1999; Chinnusamy et al., 2007; Ding et al., 2019). It is essential to unravel the molecular mechanism of cold sensing and cold stress signal transduction to avoid damage in plants caused by low temperatures. Computational models closely related to experimental observations can prove helpful to clarify the dynamics of signal transduction pathways at the cellular level. The goal of this paper will be to present such a computational model for the cold response pathway in plants.

Experimental studies in *Arabidopsis* have led to the identification of key factors in the transcriptional network of the cold acclimation pathway, where three C-repeat (CRT)-binding factors (CBFs), the most commonly studied being CBF3, also known as dehydration-responsive element (DRE)-binding proteins (DREBs), play vital roles in cold acclimation (Stockinger et al., 1997; Jaglo-Ottosen et al., 1998; Liu et al., 1998; Zhao et al., 2016). These transcription factors are induced by cold stress and bind to CRT/DRE DNA regulatory elements in the promoters of a large subset of cold-regulated (COR) genes. The expression of these *COR* genes renders plants able to tolerate freezing stress through a variety of cellular regulatory mechanisms (Thomashow, 1999; Gilmour et al., 2004; Hannah et al., 2006; Lissarre et al., 2010; Barah et al., 2013; Liu et al., 2019).

Homologues of *CBF* genes have been found in different plant species, such as the Chinese cabbage *Brassica campestris* (Wang et al., 2014), *Brassica napus* (Jaglo et al., 2001), barley (Marozsán-Tóth et al., 2015), tomato (Zhang et al., 2004), and rice (Dubouzet et al., 2003). Their functions are similar to those of the *Arabidopsis* genes, and they are also induced in response to low temperature. Given that the components of the cold response pathway are highly conserved in many plant species and that the patterns of expression of the *CBF* and *COR* genes correspond to those observed in *Arabidopsis*, the model that we propose for the cold response pathway pertains not only to *Arabidopsis thaliana* but also to other plants.

The putative temperature sensing mechanisms are able not only to respond to temperature changes but also to activate downstream response pathways (Ruelland and Zachowski, 2010). Calcium (Ca^2+^), a widely studied plant second messenger, is involved in nearly every aspect of cell physiology and development (Kudla et al., 2018). Cytosolic Ca^2+^ alterations and oscillations in plant cells, induced by a variety of environmental stimuli, are an integral component of cell signaling, and the frequency, amplitude, and spatial localization of Ca^2+^ signals control the efficiency and specificity of cellular responses (Allen et al., 2001). In plants, guard cells integrate environmental and endogenous signals to regulate the aperture of stomatal pores. Cytosolic Ca^2+^ oscillations are essential for stomatal closure, which follows cytosolic Ca^2+^ elevation in guard cell (Allen et al., 2001). Relationships between temperature sensing, specifically membrane fluidity, and Ca^2+^ signaling have been reported (McClung and Davis, 2010). The cold response in plants that follows a cold stress is strongly dependent on a fast and transient cytosolic Ca^2+^ elevation (Knight et al., 1996; Örvar et al., 2000; Cook et al., 2004).

The molecular mechanisms involved in the cold signal transduction, which include the perception of the signal, the cascade of post-translational modifications triggered by the signal, and the transcriptional regulatory network underlying cold acclimation, have all been investigated at the molecular level (Hua, 2009; Shi et al., 2018). To obtain additional insights into the dynamics of the cold response, it is useful to develop a detailed computational model that incorporates the sequence of biochemical processes involved in the signaling pathway. Such a model should eventually prove useful to interpret quantitatively the available experimental findings and to guide further experimental studies. Based on the experimental observations that revealed the structure of the pathway, we build a detailed computational model with the aim of providing a framework for a quantitative description of the regulatory network that controls the plant response to cold stress. The lack of detailed quantitative experimental observations poses a challenge in regard to parameter optimization. Most observations pertain to the time course of the expression of the gene coding for CBF3 in response to cold stress, while the time course of expression of a few other genes of the pathway has also been measured. We compare the predictions of the model with the available experimental data both in wild type and mutants.

In “The Cold Response Pathway: Brief Overview of Experimental Aspects” section, we summarize the processes underlying the response of plants to cold stress. The initial signal triggered by the cold stress takes the form of a brief influx of Ca^2+^, which rises in seconds and is over in a few minutes. This signal activates a cascade of enzymatic reactions that leads, within a few hours, to the expression of *CBF3* mRNA, which represents the major output of the pathway that is measured in experiments. Based on the regulations determined experimentally, we present, in “Modeling the Plant Response to Cold Stress” section, a computational model for the plant cold response pathway. In “Dynamics of the Response to Cold Stress: Model Predictions” section, by means of numerical simulations, we determine the dynamical evolution of the components of the network following a cold stress. The numerical results of the computational model are compared with the available experimental observations in both wild type and mutants. Under certain conditions, the model predicts the possible occurrence of an oscillatory expression of *CBF3* in response to cold stress. This potential periodic phenomenon originates from the negative feedback exerted on *CBF3* expression by ZAT12, a factor itself controlled by CBF3. An additional level of complexity arises from the control exerted by the plant circadian clock on the cold response pathway. The *CBF3* expression under natural conditions of a light-dark (LD) cycle is indeed circadian and peaks at about ZT8, i.e., 8 h after light onset, just after the early morning peak of the proteins CIRCADIAN CLOCK-ASSOCIATED 1 (CCA1) and LATE ELONGATED HYPOCOTYL (LHY) involved in the circadian clock mechanism (Dong et al., 2011). At the end of “Dynamics of the Response to Cold Stress: Model Predictions” section, we will indicate how the model for the cold response pathway can be extended to incorporate its modulation by the circadian clock, which is responsible for the gating of the plant response to cold stress. We discuss the results in “Discussion” section where we also allude to vernalization, another plant response to cold, which, in contrast to the response leading to freezing tolerance, occurs on a much longer time scale.

## The Cold Response Pathway: Brief Overview of Experimental Aspects

**Figure 1:**
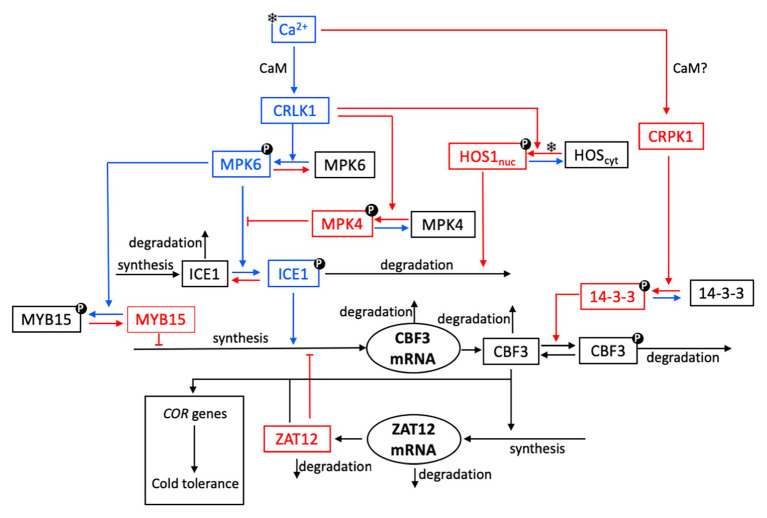
Schematic model for the cold response pathway in plants. As explained in further detail in “The Cold Response Pathway: Brief Overview of Experimental Aspects” section, the plasma membrane is thought to be the primary target of cold sensing and the starting point for transmission of the cold signal into the nucleus. The cold stress (symbolized by a snowflake) initiates a transient increase in Ca^2+^, which takes the form of a single Ca^2+^ pulse or of a train of high-frequency Ca^2+^ oscillations. The cold response pathway reacts to the cold stress by the expression of C-repeat-binding factors (CBFs, notably CBF3) that control the resistance of plants to cold stress through the expression of cold-regulated (COR) genes. For the sake of clarity, not all reaction steps are indicated in the scheme. Steps colored in blue and red denote reactions or interactions which contribute to enhance or inhibit CBF3 expression, respectively. Upon activation by calmodulin (CaM), plasma membrane Ca^2+^-regulated kinases (CRLK1/2) positively regulate cold-induced gene expression, through the MPK6 and MPK4 pathway. The cold signal activates the MPK4-MPK6 cascade to regulate freezing tolerance, through phosphorylation of ICE1. A receptor-like cytoplasmic kinase cold-responsive protein kinase 1 (CRPK1) is activated by low temperature stress and phosphorylates 14-3-3 proteins; in the absence of direct evidence (hence the question mark), we assume that this activation, as for CRLK1, is mediated by CaM (see “CBF Signaling” section). To keep the scheme simple, we do not show the active forms CRLK1a and CRPK1a resulting from the bimolecular activation of CRLK1 and CRPK1 by CaM. The key components of CBF-dependent signaling, ICE1 and CBF, are modulated through post-translational modifications. Phosphorylated MPK6 mediates the phosphorylation of ICE1, which leads to increased ICE1 degradation *via* ubiquitination; the latter process is enhanced by the high expression of the osmotically responsive gene1 (HOS1). The 14-3-3 protein kinase phosphorylates CBF3 and, thereby, facilitates the ubiquitin-mediated degradation of CBF3. Cold stress induces the accumulation of CBF proteins, which elicit the expression of ZAT12. The latter factor exerts a negative feedback on the transcription of *CBF3* mRNA.

The plant cold signaling pathway has been extensively studied in the past two decades (Chinnusamy and Zhu, 2002; Hannah et al., 2005; Thomashow, 2010; Shi et al., 2018). The salient features of the cold response pathway used in building the computational model are presented schematically in Figure 1. Below, we present in turn the messenger molecules, the protein kiases and phosphatases, and the transcription factors involved in this pathway.

### Cold Stress Sensing

Under cold shock, when the temperature drops from normal to chilling, the cytosolic concentration of Ca^2+^ ([Ca^2+^]) in plant cells shows a sharp rise before returning to a basal level. The steady-state cytoplasmic calcium concentration in plants is in the range of 80~250 nM (Knight et al., 1996). The [Ca^2+^] changes that occur during cooling are a complex function of the rate of cooling, the duration of cooling, and the magnitude of the temperature drop (Knight et al., 1996; Plieth et al., 1999). The elevation in cytosolic Ca^2+^ represents the primary signal, which is transmitted through Ca^2+^-regulated proteins, and in turn affects the phosphorylation of various proteins. The major Ca^2+^ sensor in plants in the cell plasma membrane is calmodulin (CaM), which is activated through the binding of Ca^2+^ in a cooperative manner, and ultimately regulates, *via* a signaling cascade, the expression of target genes (Zielinski, 1998). The decoding of different Ca^2+^ signatures causes the changes in gene expression that lead to the appropriate physiological responses (Knight et al., 1996; Plieth, 1999; Plieth et al., 1999).

### MAPKs Signaling

Cold stress-induced Ca^2+^ transient changes resulting in CaM activation can be decoded by different pathways. When the plant senses low temperature by an uncharacterized Ca^2+^/calmodulin-regulated receptor-like kinase (CRLK), the autophosphorylation of this CRLK —particularly CRLK1—is activated. The activation of CRLK1 leads to the phosphorylation of downstream targets such as the mitogen-activated protein kinases 6 (MPK6) and 4 (MPK4), which elicits the cold response (Yang et al., 2010; Furuya et al., 2013; Zhao et al., 2017). The MEKK1-MKK2-MPK4 cascade constitutively reduces the protein levels and kinase activities of MPK3 and MPK6 (Teige et al., 2004; Kong et al., 2012). The latter two kinases phosphorylate inducer of CBF expression 1 (ICE1), a transcription factor that regulates the expression of *CBF* genes, while the phosphorylation of ICE1 promotes its degradation (Zhao et al., 2017). The MEKK1-MKK2-MPK4 pathway constitutively suppresses MPK3 and MPK6 activities and promotes the cold response (Zhao et al., 2017).

### CBF Signaling

The expression of *CBF* genes is required for freezing tolerance in *A. thaliana* (Chinnusamy et al., 2003; Agarwal et al., 2006; Doherty et al., 2009). The expression of *CBF* genes is positively regulated by ICE1 and negatively regulated by MYB15. These transcription factors directly interact with specific elements in the *CBF* promoters. MAPK/MPK cascades function upstream to regulate *CBFs*. The downstream ZAT12 regulon appears to be involved in negative regulation of the CBF cold response pathway (Vogel et al., 2005). The *CBF3* transcript has a half-life of only 7.5 min at warm temperatures, a value that is among the shortest described for plant genes (Zarka et al., 2003).

The factor ICE1 is a major regulator that controls *CBF* expression and many other cold-responsive regulons (Chinnusamy et al., 2003). Indeed, ICE1 binds to *MYC* regulatory elements in the *CBF3* promoter and, thereby, induces the expression of *CBF3* during cold acclimation (Tang et al., 2020). The protein HOS1 negatively regulates ICE1 at low temperature by inducing its ubiquitination-mediated degradation (Dong et al., 2006). At normal growth temperature, HOS1 resides in the cytoplasm, while the nuclear localization of HOS1 is enhanced by cold stress (Ishitani et al., 1998; Lee et al., 2001). ICE1 degradation through the 26S proteasome pathway is induced by HOS1. MPK3/MPK6 phosphorylate and destabilize ICE1 and, thereby, negatively regulate *CBF* expression and the freezing tolerance in plants (Miura et al., 2007; Li et al., 2017; Zhao et al., 2017; Tang et al., 2020).

MYB15, a transcriptional repressor of cold signaling, is phosphorylated by phosphorylated MPK6 (Agarwal et al., 2006). Under normal temperatures, *CBF* transcription is repressed by the DNA-binding of unphosphorylated MYB15 to MYB recognition elements in their promoters in the absence of ICE1 activity. In response to cold stress, MYB15 is phosphorylated by cold-activated MPK6. The phosphorylated MYB15 dissociates from *CBF* promoters because of its reduced DNA-binding affinity (Kim et al., 2017).

The stability of the CBF3 protein is controlled by several processes. The kinase cold-responsive protein kinase 1 (CRPK1) and the 14-3-3 protein kinases play negative roles to prevent excessive cold responses (Liu et al., 2017). Cold-induced CBF protein accumulation is compromised by the plasma membrane CRPK1-mediated phosphorylation of 14-3-3 proteins, indicating a negative feedback function in cold signal transduction from the plasma membrane. Cold stress activates, possibly through phosphorylation by a yet unknown receptor kinase, the plasma membrane-localized protein kinase CRPK1 (Liu et al., 2017; Ding et al., 2019), which phosphorylates 14-3-3 proteins in the cytoplasm, thereby triggering 14-3-3 proteins to translocate into the nucleus. We will assume that, as for CRLK1, the activating effect of cold stress on CRPK1 is mediated by CaM. In the nucleus, phosphorylated 14-3-3 proteins promote the 26S proteasome-mediated degradation of CBF proteins, thus attenuating the CBF response. As overexpression of *CBFs* has a strong negative impact on plant growth (Achard et al., 2008), prolonged cold stress would lead to activation of a negative loop *via* CRPK1 activation. In this case, CRPK1 would ensure proper adjustment of the intensity and/or duration of the cold stress response that would need to match the intensity and/or duration of the initial cold stimulus.

## Modeling the Plant Response to Cold Stress

Our goal is to develop a detailed computational model for the plant response to cold stress involving the succession of biochemical events, from the initial Ca^2+^ influx to the rise in CBF3 that controls the expression of genes which confer tolerance to low temperatures. The model based on the experimental observations summarized above in “The Cold Response Pathway: Brief Overview of Experimental Aspects” section (where relevant references are indicated) is represented schematically in Figure 1. Arrows in blue and red denote, respectively, reactions that contribute either to enhance or to inhibit the expression of CBF3 in response to cold stress.

Starting from the top left, we can follow the reactions that are successively triggered by a cold stress. First, the decrease in temperature elicits a brief transient influx in Ca^2+^. This initial signal leads to the activation of CaM upon cooperative binding of Ca^2+^ (with a Hill coefficient of 4) to the protein. The activation of CaM brings about the activation of two protein kinases, CRLK1 (left branch) and CRPK1 (right branch). We first describe the series of reactions initiated by the activation of CRLK1. We will return afterwards to the right branch initiated by CRPK1, for which available experimental observations are not as detailed as for CRLK1.

The kinetic equations (4)–(17) governing the time evolution of the 14 state variables of the model are listed in Supplementary Material, Section 2. The list of these variables, together with their definition, are given in Supplementary Table 1, while parameter definitions and numerical values used in the numerical simulations are listed in Supplementary Table 2.

### Influx of Ca^2+^ and Activation of Calmodulin After a Cold Shock

Cold stress increases the level of cytosolic Ca^2+^ in plant cells. The rise in [Ca^2+^] takes the form of a single brief spike, or of a train of spikes. A discrete quantitative description of [Ca^2+^] dynamics for the generation of a single spike after a drop in temperature was proposed by Plieth (1999). Because we focus on the dynamics of the cold response pathway, we resort to a simpler, more straightforward approach by using an instantaneous increase followed by an exponential decrease in cytosolic Ca^2+^. Parameters were selected so as to produce a single peak in [Ca^2+^] of appropriate magnitude and duration (see Supplementary Material, Section 1.1).

Sometimes the response to a cold shock takes the form of a train of Ca^2+^ oscillations (Allen et al., 2000, 2001). To model the effect of a train of intracellular Ca^2+^ spikes, as outlined in Supplementary Material, Section 1.2, we resort to a two-variable model previously proposed for signal-induced Ca^2+^ oscillations (Goldbeter et al., 1990; Dupont et al., 1991). This widely used, generic two-variable model shows that repetitive Ca^2+^ spikes may be triggered by external stimulation, when taking into account the self-amplified release of Ca^2+^ from intracellular stores, a process known as Ca^2+^-induced Ca^2+^ release.

### The CRLK1 Branch: Promotion (and Also Inhibition) of CBF3 Production

Once activated, the protein kinase CRLK1 activates through phosphorylation the MAP kinase MPK6. As for all phosphorylation reactions considered in the model, this process is reversed by a protein phosphatase. Activated MPK6 phosphorylates and, thereby, activates the protein ICE1, which plays the major role in inducing the expression of the gene coding for CBF3. MPK6 also enhances *CBF3* expression by inactivating through phosphorylation the factor MYB15, which is a repressor of the expression of CBF3. Moreover, CRLK1 also contributes to decrease CBF3 production by activating, through phosphorylation, the MAP kinase MPK4, which inhibits the activation of ICE1 by MPK6.

An additional role of CRLK1, less documented, however, raises the possibility that this kinase, once activated by CaM, may also contribute to decrease the CBF3 response and, thus, the tolerance to low temperatures. This putative action involves the cold-stimulated entry into the nucleus and concomitant activation of the factor HOS1, which mediates the ubiquitination of ICE1 and, thereby, enhances its degradation (Dong et al., 2006).

### The CRPK1 Branch: Inhibition of CBF3

Most sensory systems display adaptation to constant stimuli (Koshland et al., 1982). After an initial response to a stimulus, the response decreases in time owing to the initiation of a mechanism that limits or inhibits the initial response. Such a phenomenon is also observed for the cold response pathway. We have already mentioned the inhibition exerted by MPK4 on the activation of ICE1 by MPK6: following its activation by CaM after the cold shock, CRLK1 indeed activates both the CBF3-enhancing MPK6 and the CBF3-inhibiting MPK4. The right branch in the scheme in Figure 1 provides an additional mechanism for the attenuation of the CBF3 response. Indeed, the Ca^2+^ influx also elicits the activation of the kinase CRPK1, likely *via* CaM. The active kinase CRPK1 phosphorylates and, thereby, activates the kinase 14-3-3 which, in turn, phosphorylates the CBF3 protein and marks it for degradation.

### Negative Feedback of ZAT12 on CBF3 Expression

The last step in the cold response pathway consists in the induction by CBF3 of *COR* genes responsible for cold tolerance. The activating effect is mediated by the transcription factor ZAT12, which is synthesized in response to a rise in CBF3. However, ZAT12 additionally acts as a repressor of the expression of the gene coding for CBF3. This regulation creates a negative feedback loop between CBF3 and ZAT12 in the lower part of the pathway (Vogel et al., 2005).

## Dynamics of the Response to Cold Stress: Model Predictions

### Dynamics of the Pathway at 20°C

We first examine the behavior of the model at a constant temperature of 20°C. At such temperature, the concentration of Ca^2+^ does not change much, so that we can treat it as constant. When the cold response pathway operates under such constant temperature, for given values of the parameters, the system converges to a unique, stable steady state. By setting the Ca^2+^ concentration to a fixed value, we can determine this steady state numerically. Linear stability analysis performed by means of the XPP program developed by Ermentrout (2002; see http://www.math.pitt.edu/~bard/xpp/xpp.html) indicates that this steady state is stable for the parameter values listed in Supplementary Table 2, which correspond to a temperature of 20°C. The Ca^2+^ steady-state level is relatively low at this temperature, so that CRLK1 is predominantly in its inactive form while MPK6, MPK4, ICE1, and MYB15 are mostly unphosphorylated. The high level of active, unphosphorylated MYB15 prevents the transcription of *CBF3* so that before the cold stress, CBF3 remains at a low stable steady-state level.

### Dynamics Under a Single Cold Shock: Response to a Single Ca^2+^ Pulse

**Figure 2:**
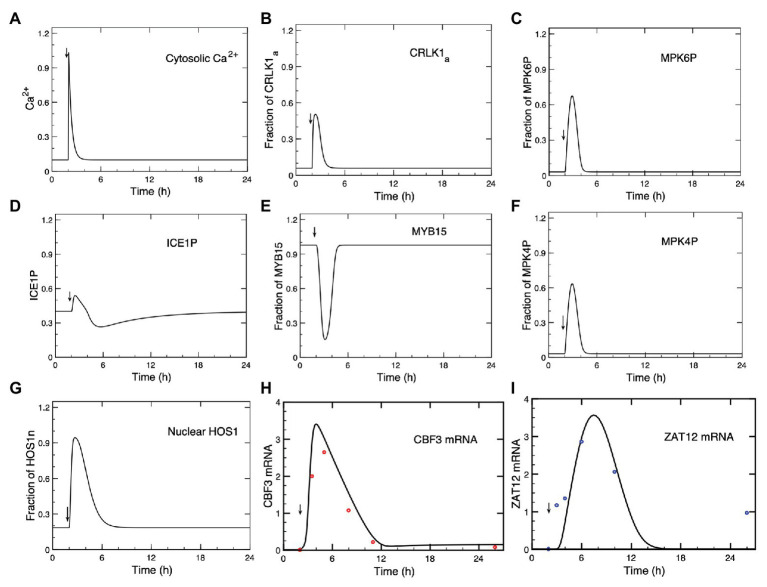
Dynamics of the cold response pathway after a cold stress. **(A)** The cold stress produces a transient increase in Ca^2+^ at time = 2 h. This brief Ca^2+^ pulse induces a transient change in the successive steps of the cascade: CRLK1a **(B)**, MPK6P **(C)**, ICE1P **(D)**, MYB15 **(E)**, MPK4P **(F)**, HOS1n **(G)**, CBF3 mRNA **(H)**, and ZAT12 mRNA **(I)**. **(H,I)** Comparison of theoretical predictions (solid lines) for the time course of CBF3 mRNA and ZAT12 mRNA with experimental data for CBF3 (red dots) and ZAT12 (blue dots) redrawn, respectively, from Figure 1 in Zarka et al. (2003) and from Figure 7A in Fowler and Thomashow (2002), by means of the software ImageJ for Image Processing and Analysis (https://imagej.nih.gov/ij/). Time is increased by 2 h when redrawing the experimental points from the original publications, because in the simulations the cold stress is given at time = 2 h (to determine the steady-state levels before the cold signal is applied) instead of 0 h in the experiments. The results are obtained by numerical integration of kinetic equations (4)–(17), by means of the program XPP (http://www.math.pitt.edu/~bard/xpp/xpp.html), for a Ca^2+^ pulse generated according to eqs. (1a–c), while the associated changes in the fraction of activated calmodulin (CaM) are determined according to eq. (3), as described in Sections 1 and 2 in Supplementary Material. Parameters are defined in Supplementary Table 2 where their numerical values are listed.

**Figure 3:**
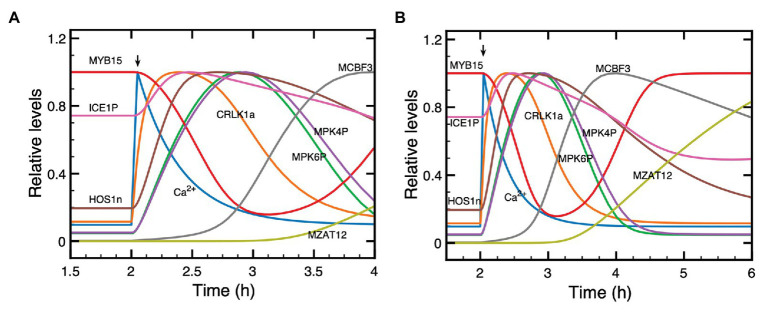
Dynamics of the cold response pathway during the first 2 h **(A)** or 4 h **(B)** after a cold stress. Shown is the time evolution of Ca^2+^, CRLK1_a_, ICE1P, HOS1n, MPK6P, MPK4P, MYB15, CBF3 mRNA, and ZAT12 mRNA. The cold signal elicits at t = 2 h a transient Ca^2+^ pulse (blue curve). Before the pulse, all state variables are at their stable steady state. To distinguish more clearly the relative positions of the different curves, each plotted state variable was normalized through division by the maximum it reaches in the course of time, since absolute levels would make it difficult to follow the order of peaks and troughs. Parameter values are the same as in Figure 2 where the time evolution is shown separately for each variable on a longer time scale.

In the model, we consider the level of *CBF3* mRNA expression as the output measuring the response to cold stress. The model allows us to follow in a detailed manner how all the elements of the cold response pathway, i.e., the state variables of the model, change in time after a drop in temperature, from the initial Ca^2+^ influx to the expression of CBF3 and ZAT12. The advantage of the model is that it permits us to follow the evolution of every state variable, as shown in Figures 2, 3, in contrast to experimental studies in which, so far, the time course has only been determined for *CBF3* mRNA and *ZAT12* mRNA. The accumulation and subsequence disappearance of these two mRNA species have been measured over the hours that follow the drop in temperature (Fowler and Thomashow, 2002; Chinnusamy et al., 2003).

In Figure 2, we show the time course predicted by the model, after the Ca^2+^ signal (A) triggered by the cold stress, for activated CRLK1 (B), phosphorylated MPK6 (MPK6P; C), phosphorylated ICE1 (ICE1P; D), MYB15 (E), phosphorylated MPK4 (MPK4P; F), nuclear HOS1 (HOS1n; G), *CBF3* mRNA (H), and *ZAT12* mRNA (I). In Figures 2H,I, we compare the time courses predicted for *CBF3* mRNA and *ZAT12* mRNA (solid curves) with experimental data (red dots for *CBF3* mRNA and blue dots for *ZAT12* mRNA) available for these state variables (Fowler and Thomashow, 2002; Chinnusamy et al., 2003).

The concentrations of CRLK1_a_ (Figure 2B), MPK6P (Figure 2C), ICE1P (Figure 2D), MPK4P (Figure 2F), HOS1n (Figure 2G), *CBF3* mRNA (Figure 2H), and *ZAT12* mRNA (Figure 2I) all increase after the cold stress, following the transient increase in Ca^2+^ (Figure 2A). Because the Ca^2+^ influx is transient, we see that the response of the network is also transient and the variables—including Ca^2+^—return to the steady state that prevailed prior to the cold stress. What is striking is the difference in time scales: while the Ca^2+^ signal rises in a few seconds and is over in a few minutes, the increase in *CBF3* mRNA peaks a few hours after the temperature drop and remains elevated for some 10 h. This represents a most significant result of our computational model, which compares well with the experimental observations, as indicated in Figure 2. Long after the concentration of Ca^2+^ has decreased, the concentrations of the other state variables of the model also decrease but this occurs on a time scale that becomes longer and longer as the effect of the brief transient signal of Ca^2+^ propagates through the successive reaction steps of the network.

As seen in Figure 2E, in contrast to the other variables plotted in the figure, the concentration of MYB15 decreases in response to the cold signal, before returning to its steady state level as Ca^2+^ decreases. When the amount of MYB15 reaches its trough, the inhibition of CBF3 decreases. As the amount of ICE1P concomitantly peaks (Figure 2D), the level of *CBF3* mRNA rises. Both the decrease in the unphosphorylated form of MYB15 and the increase in the phosphorylated form of ICE1 are brought about by their phosphorylation by MPK6P, which follows the activation of this MAP kinase by the rise in Ca^2+^ (Figure 2A).

To better grasp how the dynamics of the cold response pathway unfolds in the course of time, we plot in Figure 3 on an enlarged time scale the evolution of Ca^2+^, CRLK1_a_, ICE1P, HOS1n, MPK6P, MPK4P, MYB15, CBF3 mRNA, and ZAT12 mRNA during the first 2 h (A) and 4 h (B), after the cold stress. The Ca^2+^ pulse, triggered by the shift to cold temperatures, starts at t = 2 h. Here, plotting the different curves on the same graph allows us to clarify the sequence of activation steps that leads to *CBF3* expression in the pathway. The state variables start from their steady state levels reached before the cold stress and, after the cold signal is given, increase or decrease in response to the Ca^2+^ pulse. This pulse is followed, successively, by the peaks in CRLK1_a_, ICE1P, HOS1n, MPK6P, MPK4P, then by the trough in MYB15, and the peaks in *CBF3* mRNA and *ZAT12* mRNA. Some of the variables, such as Ca^2+^, CRLK1_a_, MYB15, ICE1P, MPK6P, and MPK4P, return more rapidly to their steady state than other variables which take more time to do so (see Figure 3B). The return to steady state of *CBF3* mRNA, followed by *ZAT12* mRNA (induced by CBF3) takes more time, because the degradation of *CBF3* mRNA proceeds at a relatively slower rate at cold temperatures.

The predicted evolution of ICE1P appears to be biphasic. First ICE1P rises and peaks in advance of its upstream variables MPK6P and MPK4P, and later decreases below steady state, due to the rise in HOS1n which promotes the degradation of ICE1P. After MPK6P, MPK4P and HOS1n reach their steady state, ICE1P begins to rise again after t = 6 h (see Figure 2D), because of the input due to constant synthesis of the ICE1 protein at a rate *v*_s1_ in eq. (10), and its conversion, activated by MPK6P, into the phosphorylated form ICE1P. This slow rise continues until ICE1P reaches its steady state. We included a term for the synthesis of ICE1 to avoid the depletion of the protein and to ensure the existence of a steady state, because we take into account the degradation of ICE1P, which is enhanced by HOS1n.

The results of Figures 2 and 3 illustrate how the model schematized in Figure 1 provides us with a detailed, integrated view of the chain of molecular events that leads from the cold-induced Ca^2+^ pulse to the synthesis of *CBF3* mRNA.

### Dynamics of the Model Under Single Cold Shock: Response to a Train of Ca^2+^ Oscillations

**Figure 4:**
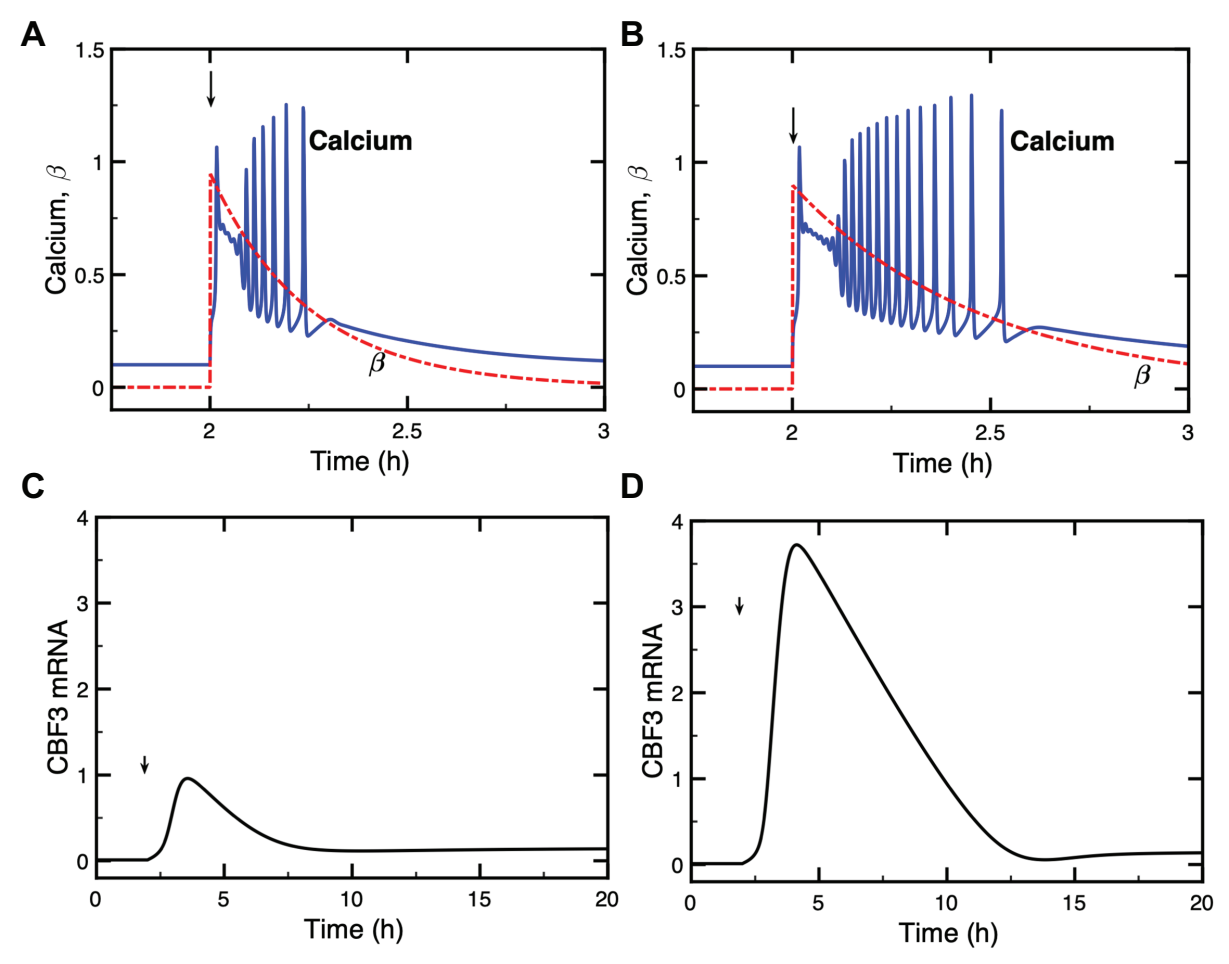
Dynamics of the model for the cold response pathway when the cold stress elicits a train of high-frequency Ca^2+^ oscillations. Panels **(A,B)**: the single cold shock elicits a train of Ca^2+^ pulses, which contains a few **(A)** or a larger number **(B)** of Ca^2+^ spikes. Panels **(C,D)** show the evolution of *CBF3* mRNA corresponding to panels **(A,B)**, respectively. After an initial increase at time t_p_ = 2 h, parameter *β* decays exponentially according to the equation $\beta =\beta_{f}\exp\left[-a\left(t-t_{p}\right)\right]$ with *β_f_* = 95% and *a* = 4 in **(A)** and *β_f_* = 90% and *a* = 2.1 in **(B)**. The curves for *CBF3* mRNA are obtained by numerical integration of the kinetic equations (4)–(17) (see Supplementary Material, Section 2) by means of XPP (http://www.math.pitt.edu/~bard/xpp/xpp.html), while the oscillatory Ca^2+^ dynamics and the associated changes in the fraction of activated *CaM* are determined as described in Supplementary Material Section 1.2, according to eqs. (2) and (3), with parameter values from Figure 2 in Dupont et al. 1991 multiplied by 25 to obtain the appropriate time scale.

In guard cells, a cold stress elicits a series of small, repetitive [Ca^2+^] transients, with an amplitude of about 125 nM and a period of about 150 s, associated with stomatal closure (Allen et al., 2000, 2001). The cold stress regulates the flow of Ca^2+^ into the cytosol, which primes the apoplast for oscillatory cycles of Ca^2+^ release. To model these oscillations so as to determine their effect on the dynamics of the cold response pathway, we assume in the model that the influx of Ca^2+^ from the apoplast is proportional to parameter *β* (red curve in Figures 4A,B) which measures the magnitude of the stimulus in the two-variable model for signal-induced Ca^2+^ oscillations (Goldbeter et al., 1990; Dupont et al., 1991).

In order to take into account the transient nature of the changes in Ca^2+^, we consider an instantaneous increase in *β* followed by an exponential decrease, which results in a transient train of Ca^2+^ oscillations (blue curves in Figures 4A,B). The expression of *CBF3* mRNA in response to this train of transient spikes is determined by numerical simulations, which demonstrate the effect of a train of Ca^2+^ oscillations (Figures 4C,D). The simulations of the model indicate that the longer the duration of Ca^2+^ oscillations, the higher the magnitude of *CBF3* mRNA accumulation in response to cold stress. The duration of the train of Ca^2+^ spikes depends on the rate of decrease in *β*, after the initial increase in this parameter brought about by the decrease in temperature.

What distinguishes the dynamics of the cold response to a single Ca^2+^ pulse or to a train of Ca^2+^ oscillations? To address this question, we compare the two situations in Supplementary Figure S1. The longer a train of oscillations, the larger the number of Ca^2+^ peaks and, therefore, the greater the magnitude of the Ca^2+^ signal acting on the cold response pathway. In panels A and C of Supplementary Figure S1, we numerically computed the area under the curve (AUC) corresponding to the oscillations in Ca^2+^ shown in Figures 4A,B; the corresponding *CBF3* mRNA peaks elicited by these oscillations are shown in Figures 4C,D, and redrawn in panels B and D in Supplementary Figure S1. Then, we generated single pulses of Ca^2+^ with comparable AUCs, as shown in panels E and G in Supplementary Figure S1. The peaks in *CBF3* mRNA produced in response to these pulses closely match those produced in response to Ca^2+^ oscillations with similar AUCs (compare in Supplementary Figure S1 panels F and H with panels B and D, respectively). It appears, therefore, that what governs the CBF3 response is the amount of Ca^2+^ liberated by the cold stress in the form of a single Ca^2+^ pulse or a train of Ca^2+^ oscillations.

### Effects of Mutations in the Cold Stress Pathway

**Figure 5:**
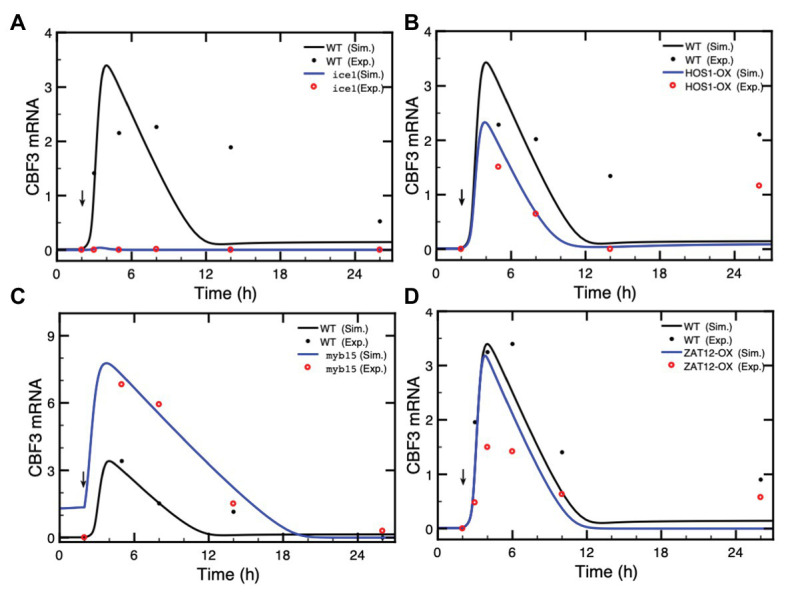
Effect of loss-of-function mutations or overexpression (OX) of genes in the cold response pathway. The expression of CBF3 is determined in the model after a cold shock given at time = 2 h (vertical arrow) in the form of a Ca^2+^ pulse. In each panel, we compare the simulated CBF3 mRNA levels in wild type (WT, black curves), and mutants (blue curves) with the CBF3 mRNA level determined experimentally in WT (black dots) and in mutants (red dots) in **(A)** the *ice1* mutant, **(B)** the overexpression of the HOS1 gene (HOS1-OX), **(C)** the *myb15* mutant, and **(D)** the overexpression of ZAT12 (ZAT12-OX). In each panel, the time at which experimental points were taken is increased by 2 h because in the simulations the cold stress is given at time = 2 h instead of 0 h in the experiments (thus a point measured at 6 h is plotted at 8 h in Figure 4B, to allow the comparison between the simulations and the experimental results). The theoretical curve (black line) obtained by simulations for the WT is the same in all panels—the changed scale in panel **(C)** is due to higher levels of *CBF3* mRNA in the *myb15* mutant—while the experimental points for the WT (black dots) are taken from different publications, as mentioned hereafter. In **(A)**, the value of ICE1 synthesis rate in the *ice1* mutant is 0.036, compared to 0.18 in WT (see Supplementary Table 2 for units of the parameters). Experimental data are redrawn, by means of the software ImageJ for Image Processing and Analysis (https://imagej.nih.gov/ij/), from Figure 1C in Chinnusamy et al. (2003). In **(B)**, the total amount of HOS1 protein in HOS1-OX is 10, vs. 2 in WT. Experimental data for CBF3 expression in HOS1-OX are redrawn from Figure 7B in Dong et al. (2006). The peak in CBF3 expression at 24 h (corresponding to time = 26 h in Figure 4B) is also observed in the wild type; see Figure 7B in Dong et al. (2006). This increase is not accounted for by the model (see “Discussion” section). In **(C)**, the total amount of MYB15 protein in the *myb15* mutant is 0.2, vs. 2 in WT. Experimental data are redrawn from Figure 7C in Agarwal et al. (2006). In **(D)**, the synthesis rate is 11 in ZAT12-OX, vs. 2.2 in WT. Experimental data are redrawn from Figure 10A in Vogel et al. (2005). Parameter values are listed in Supplementary Table 2 except for *v_s1_* = 0.036 nM/h, *v_s3_* = 11 nM/h (overexpression of ZAT12), HOS1_t_ = 10 nM, and MYB15_t_ = 0.2 nM. The results are obtained by numerical integration of kinetic equations (1) and (4)–(17) from Supplementary Material Sections 1 and 2, using the XPP program (http://www.math.pitt.edu/~bard/xpp/xpp.html).

The model of the cold response pathway allows us to predict the effect of mutations in the various elements of the network and to compare the results with available experimental data. We determine the expression of *CBF3* as the output when the levels of ICE1, HOS1, MYB15, and ZAT12 are altered as a result of mutations or overexpression. For the proteins for which the total amounts are assumed to be constant, to represent such mutations, we reduce the total amounts of MYB15 to 10% of the total amounts in wild type (WT), while we increase 5-fold the total WT concentration for HOS1 overexpression (OX) mutants. Moreover, the rate of synthesis of ICE1 is reduced to 20% to represent the effect of mutations, while the rate of synthesis of ZAT12 is increased 5-fold to represent the effect of overexpression. We compare the predictions of the model (solid lines, in black for the WT and in blue for the mutants) with experimental data (red dots) in Figure 5.

In Figure 5A, the *ice1* mutation blocks the CBF3 transcripts, in agreement with experimental observations (Chinnusamy et al., 2003), while overexpression of ICE1 (ICE1-OX) significantly enhances the expression of *CBF3* (Supplementary Figure S2) in agreement with the observed enhancement in cold tolerance (Chinnusamy et al., 2003; Lee et al., 2005). In contrast to ICE1, HOS1 is a negative regulator of low temperature-responsive gene transcription. HOS1 accumulates in the nucleus in response to low temperature treatments. The overexpression (HOS1-OX) leads to the degradation of ICE1P and represses the expression of *CBF3* mRNA (Lee et al., 2001; Dong et al., 2006). Figure 5B indicates that in the HOS1-OX mutant, the synthesis of *CBF3* mRNA is accordingly attenuated.

In contrast to ICE1, MYB15 is a repressor of *CBF3*. Following a cold stress, MYB15 is inactivated through phosphorylation, which leads to a rise in *CBF3* transcription. Overexpression of MYB15 results in the reduced expression of *CBF* genes whereas its loss-of-function leads to increased expression of *CBF* genes in the cold (Agarwal et al., 2006). Figure 5C and Supplementary Figure S3 illustrate the enhanced *CBF3* expression in the *myb15* mutant.

Finally, we turn to ZAT12, which dampens the expression of the CBF cold response pathway (Vogel et al., 2005). The overexpression of ZAT12 represses the level of *CBF3* mRNA when the level of ZAT12 protein increases above a threshold value. The expression of *ZAT12* is itself induced by CBF3. Figure 5D shows that during the first 2 h after the cold stress, the level of *CBF3* mRNA is nearly the same as in the WT because the ZAT12 protein has not yet accumulated, through induction by CBF3. Later, the higher level of the ZAT12 protein in ZAT12-OX accelerates the decrease in CBF3 because of the negative feedback exerted by ZAT12 on CBF3. As to the 14-3-3 protein, which phosphorylates CBF3, mutations in 14-3-3 reduce this phosphorylation and, hence, the expression of *ZAT12* mRNA (data not shown). The situation in regard to *CBF3* mRNA is, therefore, similar to that in *ZAT12* mutant.

### Dynamics of the Model Under Successive Cold Stresses

We focused in the previous sections on the situation where the temperature drops rapidly and produces a single transient increase in Ca^2+^ or a train of Ca^2+^ spikes. However, the temperature fluctuates seasonally, daily, as well as on a short time scale due to stochastic fluctuations. The time scale over which Ca^2+^ changes occur varies greatly, from rapid increases within seconds (Knight et al., 1996) to slower, 24 h daily rhythms (Dodd et al., 2006; Ruiz et al., 2018, 2020). Moreover, the CBF3 transcripts, in response to an instantaneous cold stress, vary during the day with a peak at about 8 h after dawn (ZT8) and with a trough at about ZT20. Cold-induced changes in *CBF3* transcription are themselves gated by the circadian clock (Fowler et al., 2005; Bieniawska et al., 2008; Nakamichi et al., 2009; Dong et al., 2011; Jiang et al., 2020), an effect to which we will return in “Circadian Gating” section below.

**Figure 6:**
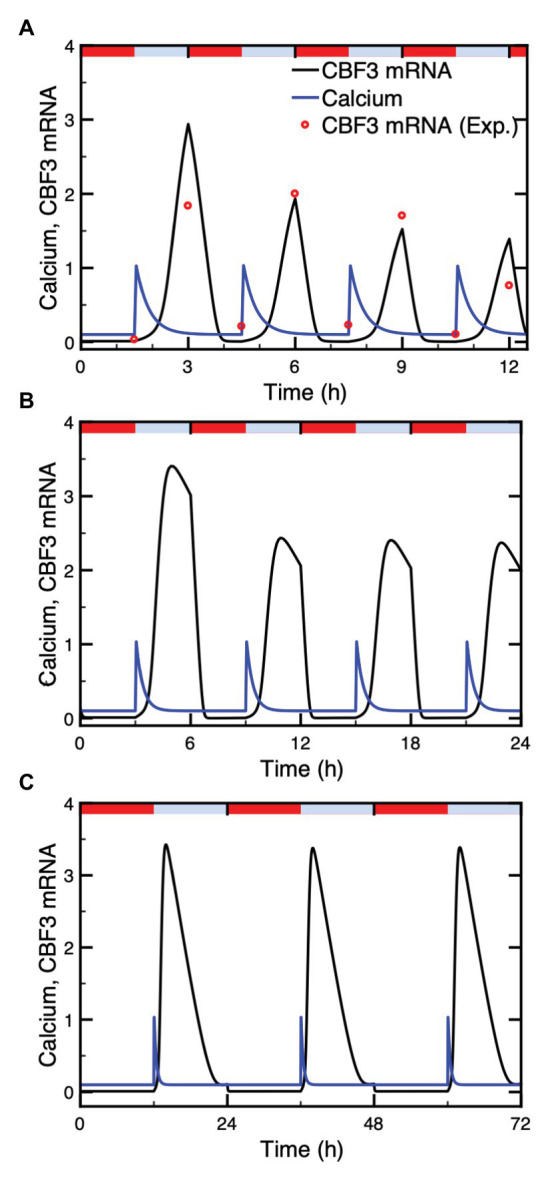
Dynamics of the cold response pathway subjected to repetitive cold stresses. Shown is the CBF3 mRNA (black curve) under identical Ca^2+^ pulses (blue curve) administered at intervals of 3 h **(A)**, 6 h **(B)**, and 24 h **(C)**. The Ca^2+^ pulses in the three panels have the same duration. Identical Ca^2+^ pulses are assumed to be elicited at the beginning of each cold phase, which is followed by a warm phase of similar duration. The duration of these phases is equal to 90 min **(A)**, 3 h **(B)**, or 12 h **(C)**. Repetitive cold stimuli are assumed to produce identical Ca^2+^ spikes. The experimental data (red points) are redrawn from Figure 2A in Zarka et al. (2003). The red bars and blue bars on top of each panel represent warm (20°C) and cold (4°C) phases, respectively. Parameter values are listed in Supplementary Table 2. The results are obtained by numerical integration of eqs. (1) and (4)–(17), and by means of eq. (3) for the determination of *CaM* (see Supplementary Material Sections 1 and 2), using the XPP program. The degradation rate of CBF3 mRNA is 0.55 and 5.5 in cold and warm phases, respectively.

The dynamics of the cold response pathway under successive cold stresses has been studied experimentally by Zarka et al. (2003). Their observations allow us to compare the predictions of the model with the results they obtained in such conditions. We represent the warm-cold cycle by a series of identical Ca^2+^ pulses and take into account the increased rate of *CBF3* mRNA degradation at higher temperatures. The time evolution of *CBF3* mRNA under different warm-cold cycles is presented in Figure 6. We tested 3 cycles characterized by a period of 3 h (90 min cold followed by 90 min warm, as in the experiments of Zarka et al., 2003; Figure 6A), 6 h (Figure 6B), and 24 h (Figure 6C), respectively. In each warm-cold cycle, we assume that Ca^2+^ increases transiently at the beginning of the cold interval and that the degradation rate of *CBF3* mRNA rises 10-fold in the warm phase.

Numerical simulations indicate that the *CBF3* mRNA level, which increases in response to the cold stress and diminishes due to both the end of the Ca^2+^ pulse and the enhanced degradation at higher temperatures, undergoes periodic variations, in phase with the warm-cold cycles. The level of *CBF3* mRNA under 3 h-warm-cold cycles displays desensitization of the cold response (Zarka et al., 2003), since the amplitudes of *CBF3* mRNA in each cycle gradually declines (Figure 6A). Under natural circumstances, the temperature fluctuates daily with, typically 12 h warm and 12 h cold. Figure 6C shows that the amplitude of *CBF3* mRNA then remains the same in each cycle. This is due to the decay of downstream inhibitors, such as ZAT12, in the course of time, which allows CBF3 to return to high levels. In Figure 6B, the amplitudes of *CBF3* mRNA in 6 h cycles drops but significantly less compared to the 3 h cycles. This suggests that the time to restore the transcription rate at maximum level is about 12 h. The corresponding value shown by the data of Figure 2B in Zarka et al. (2003) is between 8 and 24 h.

### Putative CBF3 Oscillations Resulting From Negative Feedback Exerted by ZAT12

**Figure 7:**
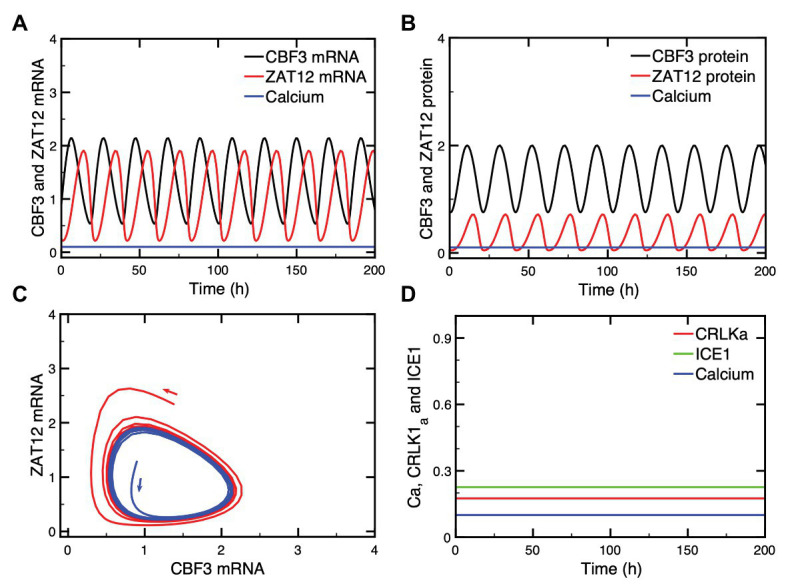
Oscillations in CBF3 expression in the absence of a cold shock. In some conditions, oscillations can occur spontaneously in some state variables of the cold response pathway, such as CBF3 and ZAT12, both in their mRNAs **(A)** and protein levels **(B)**. When the levels of CBF3 and ZAT12 mRNAs are plotted against each other in the course of time, the oscillations correspond to the evolution toward a closed curve known as a limit cycle **(C)**. This limit cycle can be reached starting from different initial conditions, as shown by the trajectories in blue and red in **(C)**. By contrast, other state variables located upstream of CBF3 in the cold response pathway do not oscillate, as shown in **(D)** for CRLK1a (red curve) and ICE1 (green curve) which remain at a stable steady state, in the absence of cold stress. The Ca^2+^ level (blue curve) accordingly remains also at a stable steady state level. The curves are obtained by numerical integration of eqs. (1), (3), and (4)–(17) from Supplementary Material Sections 1 and 2. Parameter values are listed in Supplementary Table 2 except for the following parameters: *K*_a2_ = 0.55, *K*_I2_ = 0.8, *K*_I3_ = 0.2, *K*_d3_ = 1.8, *k*_s1_ = 0.25, *v*_d5_ = 3.8, *v*_s3_ = 3.5, *K*_a3_ = 0.8, *v*_d6_ = 3.2, *k*_s2_ = 0.55, *v*_d7_ = 3.65, and *k*_d7_ = 0.1 (see Supplementary Table 2 for the definitions and units of these parameters).

The investigation of the dynamics of the model for the plant response to cold stress has revealed, unexpectedly, the possibility of sustained oscillations in the expression of *CBF3*. This phenomenon, which occurs spontaneously for a restricted set of parameter values, does not need to be triggered by the Ca^2+^ influx that occurs upon decreasing temperature to a low level. Such oscillations are shown in Figures 7A,B for the mRNA and protein levels, respectively, both for CBF3 and ZAT12. Projecting the trajectory onto a phase plane formed by *CBF3* mRNA and *ZAT12* mRNA indicates that this periodic behavior corresponds to the evolution toward a closed curve known as limit cycle (Figure 7C). The conspicuous property of the limit cycle is that it can be reached regardless of initial conditions and, therefore, represents a very robust mode of oscillations. The other state variables upstream in the plant cold response pathway (see Figure 1) evolve to a stable steady state after the cold stress. Thus, the oscillations in CBF3 and ZAT12 are not observed in other proteins involved in the cold response pathway, as shown in Figure 7D.

What is the origin of such CBF3 oscillations? Negative feedback represents a well-known source of oscillatory behavior in biochemical systems at the cellular level, as exemplified by the molecular mechanism of the circadian clock, which involves auto-regulatory negative feedback loops on gene expression in a variety of organisms, including *Drosophila*, mammals, and plants (Goldbeter, 1995; De Caluwé et al., 2016). Many other examples of oscillations originate from regulatory feedback processes at the cellular level (see Goldbeter, 1996; Goldbeter et al., 2012, for reviews). Here, a negative feedback is exerted by ZAT12 on the expression of *CBF3*. Indeed, the expression of *ZAT12* is induced by CBF3 while ZAT12 represses the expression of *CBF3*. This negative feedback regulation is responsible for the oscillatory expression of CBF3, which is characterized by a period of about 32 h for the parameter values considered in Figure 7. The characteristics of these oscillations are further explored in Supplementary Material, Section 4 and in Supplementary Figure S4.

That the mechanism of the oscillations relies on the negative feedback exerted by ZAT12 on *CBF3* expression is shown by the observation that while CBF3 and ZAT12 oscillate, the other state variables located upstream in the cold response pathway (see Figure 1) do not display oscillations and remain at a stable steady state, as illustrated in Figure 7D. This difference is due to the fact that the regulation exerted by ZAT12 at the last step of the pathway schematized in Figure 1 does not affect the synthesis or degradation of the state variables involved in the preceding steps of the network.

What are the conditions that could favor the onset of oscillations in CBF3 expression? The stability diagram in Supplementary Figure S4D suggests that oscillations might be induced by decreasing the rate of degradation of *CBF3* mRNA, *v*_d3_, when the system moves first from the stable steady state P_2_ to the stable steady state P_3_ (in which the level of *CBF3* mRNA is higher, as can be seen in Supplementary Figures S4E,F, because of the reduced rate of degradation) and then to the unstable steady state P_3_ around which sustained oscillations occur. The same diagram in Supplementary Figure S4D shows that oscillations could also result from increasing the maximum rate of *CBF3* expression *v*_s2_ at a given value of *v*_d3_. The model predicts, however, that other parameter changes may also induce oscillations, for example—as suggested by the diagram in Supplementary Figure S4B—by increasing (on the left of this diagram) or decreasing (on the right of this diagram) the maximum rate of ZAT12 mRNA degradation, *v*_d6_, or by increasing the ratio *k*_9_/*v*_5_ which measures the ratio of phosphorylation and dephosphorylation rates of ICE1. Increasing this ratio favors the rise in ICE1P, which enhances the expression of *CBF3* (see scheme of the model in Figure 1). In conclusion, all parameters which directly or indirectly enhance the formation of CBF3, by increasing its synthesis or decreasing its degradation, favor the onset of oscillations, provided that the level of ZAT12 is in an appropriate range, as shown in Supplementary Figure S4B.

### Circadian Gating

Many aspects of plant physiology and metabolism are controlled by the circadian clock. Unsurprisingly, the cold response was also shown to be under the circadian control (Fowler et al., 2005; Dong et al., 2011). Circadian rhythms are generated at the cellular level by a dozen of clock genes, which form a network of interlocked regulatory feedback loops (Nohales and Kay, 2016; McClung, 2019). This genetic clock is entrained by the external light-dark cycle and controls the expression of many genes, including *CBF* genes (Fowler et al., 2005; Dong et al., 2011). Moreover, the circadian phase at which plants were transferred to low temperature affects the level at which *CBF* transcripts accumulate upon cold stress (Fowler et al., 2005). That the induction of *CBF* genes is gated by the circadian clock was corroborated by subsequent experiments (Dong et al., 2011; Keily et al., 2013).

**Figure 8:**
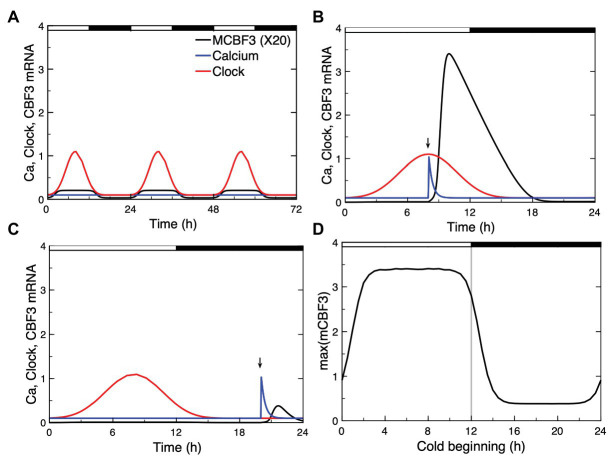
Circadian gating of the cold response. Depending on the time of the day at which the cold stress is applied, CBF3 expression can be strongly induced or not. **(A)** In the absence of cold stress, the Ca^2+^ level (blue curve) remains at a stable steady state, while CBF3 mRNA undergoes small amplitude oscillations (black curve) driven by the circadian clock (red curve). The 12 h light (L)-12 h dark (D) LD cycle is represented on top of the figure in such a way that the maximum in CBF3 mRNA occurs at ZT8, i.e., 4 h before the L to D transition, as observed experimentally (Dong et al., 2011). For the sake of clarity, the level of CBF3 mRNA in **(A)** is multiplied by 20. **(B)** When the cold stress, resulting in a brief Ca^2+^ pulse, is applied at a phase when CBF3 driven by the circadian clock is near its maximum, the cold stress results in a large-amplitude peak in CBF3 produced by the plant cold response pathway. **(C)** By contrast, when the Ca^2+^ pulse is applied at a phase when CBF3 is away from its maximum, the cold stress fails to elicit the activation of the plant cold response pathway. **(D)** Plotting the maximum of the CBF3 response as a function of the time at which the same Ca^2+^ pulse is applied shows that the circadian clock defines a window of time during which a significant cold response can be achieved. The curves are obtained as described for Figure 2, for the parameter values listed in Supplementary Table 2. The circadian oscillation that controls CBF3 expression is generated according to eqs. (18) and (19) in Supplementary Material Section 3, with *K*_c_ = 0.15 and *C*_0_ = 0.01.

To account for this circadian gating in our model, we simulated the circadian clock signal by a sine function (see Supplementary Material, Section 3). This circadian variable, which could represent the level a circadian clock protein (e.g., CCA1/LHY), acts at the level of *CBF3* expression, as expressed by eqs. (18)–(19); (see Supplementary Material, Section 3). In Figure 8A, we show the circadian variation in *CBF3* expression controlled by the circadian clock in the absence of cold stress. Although the LD cycle is not modeled explicitly in this work, we add for clarity the 12 h:12 h LD cycle on top of the panel, with a phase set so that the peak in *CBF3* mRNA occurs at ZT 8, as observed experimentally (Dong et al., 2011).

Our simulations show that, depending on the time at which the Ca^2+^ pulse triggered by the cold stress is applied, *CBF3* expression can be strongly induced (Figure 8B) or not (Figure 8C). The responsiveness to cold stress is shown in Figure 8D as a function of the timing of the Ca^2+^ pulse initiated by the cold stress. These results support the view that the circadian clock limits the cold response to a certain window of time.

## Discussion

The aim of this study was to build a detailed computational model of the pathway responsible for the plant response to cold stress, based on available experimental observations. Our work provides a theoretical framework that we used to address the dynamics of this important regulatory network in a variety of conditions. The model takes into account the results from numerous biochemical and genetic studies which showed how a cold stress activates a signaling cascade leading to the expression of *COR* genes, which enable plants to tolerate freezing temperatures (Thomashow, 1999; Gilmour et al., 2004; Hannah et al., 2006; Lissarre et al., 2010). The cold stress triggers a signal in the form of a brief Ca^2+^ spike, initiating a cascade of biochemical reactions that eventually lead to a rise in the factor CBF3, which induces the expression of *COR* genes after several hours. As shown in Figures 2 and 3, the model allows us to follow how the initial signal is transduced through successive steps which eventually produce the rise in CBF3. Experimentally, the plant response to cold stress is measured by the level of *CBF3* mRNA transcripts which, in the wild type, peaks between 1 and 6 h after cold stress, according to different experiments (Fowler and Thomashow, 2002; Chinnusamy et al., 2003). In the model, we consider that the brief Ca^2+^ pulse represents the initial response to the cooling stimulus. Remarkably, this initial Ca^2+^ pulse, which rises in seconds and is over in minutes, results in the sequential activation of various protein kinases, and finally in the transient expression of genes over a much longer time scale, of the order of hours.

The model allows us to follow *in silico* the detailed chain of biochemical events that leads from the cold-induced Ca^2+^ signal to *CBF3* expression. As done in Figures 2, 3, we can use the computational model as a magnifier to enlarge the details of the time evolution of the different variables in the model so as to disentangle their time course in the minutes and hours that follow the cold stress. Obtaining experimentally such detailed comparative information would be extremely valuable. The predictions of the computational model provide us with a framework that may prove useful to this end.

Because of the many regulatory interactions that link the biochemical variables in the cold response pathway schematized in Figure 1, the detailed sequence of events leading to *CBF3* expression is sometimes difficult to predict in the absence of a computational model. Thus, in the chain of sequential activations, as shown in Figure 3, once activated by CaM CRLK1a activates the kinases MPK6 and MPK4 which exert antagonistic effects on ICE1P. Moreover, CRLK1 also activates HOS1 which controls the degradation of ICE1P. The time course of ICE1P is, therefore, controlled by the combined effects of MPK6, MPK4, and HOS1. Similarly, the time evolution of *CBF3* mRNA is activated by ICE1P and inhibited by MYB15 and ZAT12, the latter being induced by CBF3. The model allows us to predict the time course of all these variables as it takes into account and integrates simultaneously all regulatory interactions in the pathway. It might be difficult to reach such a comprehensive, global view of the dynamics of the cold response pathway without a computational approach.

Rather than eliciting a single Ca^2+^ spike, the cold stress sometimes triggers a train of high-frequency Ca^2+^ oscillations (Allen et al., 2001). We determined the effect of such an oscillatory signal on the response of the model for the cold response pathway using a generic model for Ca^2+^ oscillations based on the Ca^2+^-induced Ca^2+^ release mechanism (Goldbeter et al., 1990; Dupont et al., 1991). As shown in Figure 4, because it occurs on a much longer time scale the expression of *CBF3* integrates the high-frequency Ca^2+^ spikes without reflecting each of them. The amplitude of the response thus increases with the number of spikes and, hence, with the duration of the Ca^2+^ oscillatory signal.

We used the model to predict the effect of mutations or overexpression of various genes in the cold response pathway. Every step in the cascade leading from the initial Ca^2+^ pulse to *CBF3* expression is capable of affecting the response to cold stress. As shown in Figure 5 and in Supplementary Figures S2, S3, the predictions of the model agree with experimental observations as to how the expression of *CBF3* is affected by mutations in or overexpression of various genes such as those coding for ICE1, HOS1, MYB15, or ZAT12. In Figure 4B, established for HOS1-OX, the experimental data for *CBF3* expression (red dots) display a peak at 24 h (corresponding to time = 26 h in Figure 4B, as explained in the legend to Figure 4). This peak is also observed in the wild type; see Figure 7B in Dong et al. (2006). Such increase, which is not accounted for by the simulations of the model is, however, not always observed in the experiments; see, for example, Figure 2H in Zarka et al. (2003). It might perhaps be due to another mechanism, such as circadian gating, considered in “Circadian Gating” section.

Experimental observations indicate that *CBF3* transcription displays desensitization and resensitization in response to successive cold stresses (Plieth et al., 1999; Zarka et al., 2003). Both for Ca^2+^ and *CBF3* transcripts, when the decreasing temperature stimuli are repeated, the amplitudes of the [Ca^2+^] spikes and *CBF3* expression levels decrease (Plieth et al., 1999). The dependence on past low temperature episodes that the plant previously encountered indicates that the [Ca^2+^] spikes and *CBF3* transcript response to cooling or in response to a stepwise decrease of the temperature can be attenuated (Knight et al., 1996; Zarka et al., 2003). The cold-sensing mechanism resensitizes after 8–24 h at warm temperatures (Zarka et al., 2003). The results of numerical simulations of the model indicate that even in the case where the system is subjected to a series of identical Ca^2+^ pulses corresponding to repetitive alternations of cold and warm phases, the cold response pathway is capable of displaying by itself desensitization (Figure 6A). The phenomenon attenuates (Figure 6B) and finally disappears (Figure 6C) when the interval between successive cold phases increases sufficiently.

As with all models involving a large number of state variables and parameters, a major question arises in regard to parameterization, given that quantitative information on many parameters in such models is often scarce, if not unavailable. As exemplified by more complex models involving even greater numbers of state variables and parameters, such as those proposed for the circadian clock (Leloup and Goldbeter, 2003) or the mammalian cell cycle (Gérard and Goldbeter, 2009), the computational models for cellular regulatory pathways remain useful for studying the mechanism and dynamics of these processes at the molecular and systems levels, and sometimes to predict new, counter-intuitive modes of qualitative behavior, even though precise parameter values are lacking in these models. One way of dealing with the issue of parameterization is to study numerically the dynamic behavior of the model over large ranges of parameter values. In addition, a sensitivity analysis may be performed to investigate the range of parameter values in which certain experimental observations can be reproduced (see, for example, Leloup and Goldbeter, 2004; De Caluwé et al., 2016).

As explained in detail in Section 4 of Supplementary Material, both approaches were followed in this study of the model for the cold response pathway, which contains 14 state variables and 77 parameters, not counting those related to Ca^2+^ and CaM. Most of these parameter values were not (yet) characterized experimentally. For example, kinetic studies have yet to be performed to yield values for the various rates of phosphorylation or dephosphorylation, for the Michaelis constants that characterize these enzyme reactions, for the activation or inhibition constants that measure the regulatory interactions, and for the total concentrations of the different molecular species involved in the pathway. For all these parameters, we made a semi-arbitrary choice, in a physiologically reasonable range, as explained in Section 4.1 of Supplementary Material.

The choice of parameter values was guided by the constraint of matching the observed characteristics of the *CBF3* expression, which is the major response measured in the experiments, in regard to its amplitude, timing, and half-width. This was achieved by trial and error, i.e., by performing simulations for a large set of parameter values and selecting a set that produced (1) a *CBF3* mRNA peak in a range extending from 1 to 6 h after the cold stress, as observed in different experiments (Chinnusamy et al., 2003; Zarka et al., 2003; Vogel et al., 2005; Agarwal et al., 2006; Medina et al., 2011); (2) an amplitude in the range of about 20–500 fold-increase compared to the steady state level prior to the stress (Chinnusamy et al., 2003; Zarka et al., 2003; Dong et al., 2011); and (3) a range of 3–6 h for the half-width of the peak in *CBF3* expression induced by the cold signal. For the brief initial Ca^2+^ pulse, we chose values for the kinetic parameters ensuring that the pulse is over in minutes, as observed in the experiments (Plieth et al., 1999). For Ca^2+^ oscillations, we selected parameter values in eqs. (2a)–(2d) as described in Section 1.2 of Supplementary Material, so as to obtain a period of the order of 150 s as observed in plant guard cells (Allen et al., 2001). The degradation rate of *CBF3* mRNA has been determined experimentally. Thus, the *CBF3* transcript has a half-life of 7.5 min at warm temperatures, which gives an approximately degradation rate of *CBF3* mRNA at 20°C of the order of 5.5 nM/h, while at low temperature this degradation rate is 10 times smaller, of the order of 0.55 nM/h (Zarka et al., 2003). This quantitative information was taken into account in numerical simulations of the model.

We also performed a sensitivity analysis, as detailed in Section 4.2 in Supplementary Material, to determine for each parameter, varying one at a time, the range in which the model matches experimental observations with respect to three properties of the peak of *CBF3* expression elicited by a cold stress: the amplitude of the peak in *CBF3* mRNA, the timing of this peak, and the half-width, measuring the duration of the response. This analysis allows us to determine which parameters might be targeted experimentally to modify most effectively the amplitude, timing, or half-width of the peak in *CBF3* expression elicited by the cold stress.

An additional interest of the computational model is to reveal the possibility of new modes of behavior, such as oscillations in *CBF3* expression, which, so far, have not been observed experimentally, and to investigate the effect of coupling the plant cold response pathway to the circadian clock. In a certain range of parameter values, the model indeed predicts the possible occurrence of sustained oscillations in the expression of *CBF3*. The phenomenon is accompanied by oscillations in ZAT12 but not in the other state variables of the network. The mechanism of CBF3 oscillations involves the negative feedback exerted by ZAT12 on the expression of *CBF3*. Numerical simulations indicate, as shown in “Putative CBF3 Oscillations Resulting From Negative Feedback Exerted by ZAT12” section and Supplementary Figure S4, that oscillations are favored by a high level of phosphorylated ICE1, which acts as inducer of *CBF3*. Besides augmenting the stability of *CBF3* mRNA, enhancing the level of phosphorylated ICE1 might, therefore, be a promising approach for investigating the possibility of an oscillatory expression of the genes that control the plant response to cold stress. The possible physiological significance of such oscillations in *CBF3* expression and their very occurrence nevertheless remain an open question, given that they only occur for appropriate parameter values and that so far they have not been observed experimentally. Besides these ZAT12-controlled oscillations, the cold response pathway is subjected to periodic alternations between cold and warm periods and to control by the endogenous plant circadian clock, which appears to gate the plant response to cold stress, as shown in “Circadian Gating” section and Figure 8. Because of their tight coupling through CBF3, ZAT12-controlled oscillations, if they occur, might well synchronize with these endogenous and exogenous circadian variations.

Incorporation of the effect of the circadian clock on *CBF3* expression into the model allowed us to account for circadian gating of the response to a drop in temperature. We represented the circadian input phenomenologically by means of a sine function multiplying the rate of *CBF3* mRNA synthesis. It would be possible to replace this description by using a compact model for the plant circadian clock (De Caluwé et al., 2016) in which CCA1 and LHY are treated explicitly as variables. This would allow us to test more directly the effect of different photoperiods as well as the effect of mutations in clock genes on the plant response to cold stress. In support of experimental observations (Fowler et al., 2005; Dong et al., 2011), the results of Figure 8 indicate that the operation of the cold response pathway is gated by the circadian clock. Experimentally, the maximum expression of *CBF3* occurs during the day, several hours before the L to D transition. This timing does not correspond to the time at which the cold stress is expected to occur in physiological conditions, which should be in late evening or at night. The same remark holds for the results of Figure 8, which indicate that in the model also the window of maximum responsiveness of the cold response pathway is largely confined to the L phase. The results predict that in a portion of the LD cycle, the reaction of the plant cold response pathway to a Ca^2+^ pulse is negligible. From a physiological point of view, we would not expect the cold response pathway to operate optimally if the cold stress arrives at such phases. In discussing the relative timing of the system’s response with respect to the cold stress, we should keep in mind that a delay likely exists between the rise in *CBF3* expression and the build-up of the cellular response to cold stress. A large number of *COR* genes must indeed be induced by CBF3, and their translation into the corresponding proteins takes time, while additional delays should arise from the time taken by these proteins to organize cold acclimation and freezing tolerance at the cellular level.

Besides the pathway responsible for resistance to a cold stress, plants display a separate cold-dependent response, which occurs on a time scale of weeks or months. This major cold-dependent physiological process, known as vernalization, is the phenomenon by which prolonged cold exposure leads to the silencing of a floral repressor gene (Song et al., 2012), thereby allowing the plant to delay its flowering until after the winter season. This epigenetic process has been modeled mathematically in terms of bistability in the flower-control regulatory system (Angel et al., 2011). Here, we focused on modeling the rapid response of plants to a cold stress that leads, after a few hours, to freezing tolerance. Although they appear to be largely independent and occur on widely different time scales, some experimental observations provide evidence (Diallo et al., 2010; Feng et al., 2017) for cross-talk between the two cold-dependent signaling pathways.

## Data Availability Statement

The original contributions presented in the study are included in the article/Supplementary Material, further inquiries can be directed to the corresponding authors.

## Author Contributions

XY, XH, and AG designed the study. RZ, AG, and DG built the computational model. RZ and DG performed numerical simulations. RZ and AG wrote the manuscript. RZ, DG, XH, XY, and AG contributed to discussing the results and to finalizing the manuscript. All authors contributed to the article and approved the submitted version.

### Conflict of Interest

The authors declare that the research was conducted in the absence of any commercial or financial relationships that could be construed as a potential conflict of interest.
