# Supplementary material for: A Computational Model for the Cold Response Pathway in Plants
Source: Front Physiol. 2020 Nov 5;11:591073. doi: 10.3389/fphys.2020.591073 (PMC7674828; doi:10.3389/fphys.2020.591073)
Supplement: Supplementary file 1 [file Data_Sheet_1.pdf]

A computational model for the cold response pathway in plants

Supplementary Material

Page

1. **Modeling the Ca^{2+} signal triggered by the cold stress** …………………….…..…… 2
   1.1. Single Ca^{2+} pulse………………….…………………….….…….…………… 2
   1.2. Ca^{2+} oscillations…..…………………….………………………...………… 2
2. **Kinetic equations for the cold stress response pathway**………………….……….. 4
3. **Circadian gating**…………………………….………………………………... 8
4. **Model parameterization and sensitivity analysis**……………………………....…. 8
5. **Supplementary Figures and Tables**…………………………………………….. 14
   Figure S1: Pulsatile versus oscillatory Ca^{2+} signal………………..……...……. 14
   Figure S2: Mutation or overexpression of ICE1……………….….……………… 16
   Figure S3: Mutation of MYB15……….………………..…………………………. 17
   Figure S4: ZAT12-induced oscillations in CBF3 expression……………………...... 18
   Figure S5: Sensitivity for amplitude, timing and half-width of *CBF3* mRNA peak….. 19
   Figure S6: *CBF3* expression without or with oscillations….…….….…………….. 21
   Figure S7: Sensitivity analysis: Dependence on initial conditions………..…….…… 22
   Supplementary Table 1: Variables of the model…...……………….…………… 24
   Supplementary Table 2: Parameter definitions and numerical values……………..…. 25
   Supplementary Table 3: Sensitivity with respect to peak of *CBF3* expression……..... 29
   Supplementary Table 4: Sensitivity with respect to peak timing.………….........…… 31
   Supplementary Table 5: Sensitivity with respect to half-width of peak………………. 33
6. **Replotting data from experimental figures** ……………….………..………..…….. 35
7. **Computer codes for numerical simulations**……………………...…………..… 36
8. **References cited in Supplementary Material**…………………………………….. 43

1. Modeling the Ca^{2+} signal triggered by the cold stress

1.1 Single Ca^{2+} pulse

The first step of the modeling pertains to the Ca^{2+} influx triggered by the drop in temperature. Plieth (1999) proposed a detailed mathematical description of this process based on the observation that the Ca^{2+} influx into the cytoplasm is mediated by calcium-permeable channels which are assumed to be solely dependent on the cooling rate, while the Ca^{2+} efflux is mediated by Ca^{2+} pumps which depend on the temperature. This model reproduces the experimentally observed features of the cooling-induced changes in the Ca^{2+} concentration $[Ca^{2+}]$ but proved difficult to handle numerically. Therefore, we resorted to a simple phenomenological description of the Ca^{2+} pulse as an instantaneous increase followed by an exponential decrease in $[Ca^{2+}]$. The time evolution of $[Ca^{2+}]$ is governed by eq. (1a)

$$\frac{\mathrm{d}[\mathrm{Ca}^{2+}]}{\mathrm{d}t} = I_{\mathrm{IN}} - I_{\mathrm{EX}} \tag{1a}$$

where I_{IN} and I_{EX} denote, respectively, the Ca^{2+} influx into the cell and the Ca^{2+} efflux. For simplicity, we take the influx as a pulse of magnitude I_{INmax} and duration Θ_{drop} superimposed on a basal influx I_{IN0}.

$$I_{\mathrm{IN}} = I_{\mathrm{IN0}} + I_{\mathrm{INmax}}\Theta_{\mathrm{drop}} \tag{1b}$$

where $\Theta_{\mathrm{drop}} = 1$ when temperature drops, 0 otherwise. We consider that the efflux is proportional to the Ca^{2+} concentration:

$$I_{\mathrm{EX}} = I_{\mathrm{EXmax0}}[\mathrm{Ca}^{2+}] \tag{1c}$$

In the simulation, we take the values of I_{IN0}, I_{INmax} , I_{INmax0} as equal to 0.28, 20, and 2.8, respectively. The duration for each cold-triggered Ca^{2+} influx, Θ_{drop}, is 0.05 h, i.e., 3 min.

1.2 Ca^{2+} oscillations

The cold stress sometimes triggers a train of high-frequency $[Ca^{2+}]$ oscillations (see Section 4.3 in main text). To model these oscillations we resort to a two-variable model for signal-induced oscillations of intracellular Ca^{2+} based on stimulus-induced synthesis of inositol-triphosphate ($InsP_3$), $InsP_3$-induced Ca^{2+} release from an InsP3-sensitive intracellular Ca^{2+} pool, and Ca^{2+}-induced Ca^{2+} release from an $InsP_3$-insensitive Ca^{2+} pool (Goldbeter et al., 1990; Dupont et al., 1991). The synthesis of $InsP_3$ is assumed to be elicited by the stimulus (here, the cold stress). Cytosolic Ca^{2+} activates the flux of Ca^{2+} from the $InsP_3$-insensitive pool

into the cytosol. This Ca^{2+}-induced Ca^{2+} release (CICR) process plays a primary role in the onset of oscillations.
The time evolution of two variables, concentration of free Ca^{2+} in the cytosol (denoted as Z) and in the InsP3-insensitive pool (denoted as Y), is governed by kinetic equations:

$$\frac{\mathrm{dZ}}{\mathrm{dt}} = v_0 + v_1\beta - v_2 + v_3 + k_\mathrm{f}\mathrm{Y} - k\mathrm{Z} \quad (2a)$$

$$\frac{\mathrm{dY}}{\mathrm{dt}} = v_2 - v_3 - k_\mathrm{f}\mathrm{Y} \quad (2b)$$

with

$$v_2 = V_{\mathrm{M2}}\frac{\mathrm{Z^n}}{K_2^\mathrm{n}+\mathrm{Z^n}} \quad (2c)$$

$$v_3 = V_{\mathrm{M3}}\frac{\mathrm{Y^m}}{K_\mathrm{R}^\mathrm{m}+\mathrm{Y^m}}\cdot\frac{\mathrm{Z^p}}{K_\mathrm{A}^\mathrm{p}+\mathrm{Z^p}} \quad (2d).$$

In these equations, v_0 is the constant input of Ca^{2+} from the extracellular medium; $v_1\beta$ is the InsP3-modulated release of Ca^{2+} from InsP3-sensitive store. The rate v_2 refers to the pumping of Ca^{2+} into InsP3-insensitive store; the rate v_3 refers to the release of Ca^{2+} from InsP3-insensitive store into the cytosol, activated by cytosolic Ca^{2+}; V_{M2} and V_{M3} denote the maximum values of these rates. Parameters K_2, K_R and K_A are threshold constants for pumping, release and activation; k_f is a rate constant measuring the passive, linear leak of Y into Z; k relates to the assumed linear transport of cytosolic Ca^{2+} into the extracellular medium. The amount of released Ca^{2+} is controlled by the level of external stimulus through modulation of saturation function of β the InsP3 receptor.

Oscillations in cytosolic Ca^{2+} arise either spontaneously or in an appropriate range of external stimulation. To obtain the period of Ca^{2+} oscillations observed in plant guard cells, which is of the order of 150 s with an amplitude of the order of 125 nM (Allen et al., 2001), we take parameter values based on values from Dupont et al. (1991), where $v_0 = 25$ µM.h^{-1}, $v_1 = 182.5$ µM.h^{-1}, $V_{\mathrm{M2}} = 1625$ µM.h^{-1}, $V_{\mathrm{M3}} = 12500$ µM.h^{-1}, $K_\mathrm{R} = 2$ µM, $K_\mathrm{A} = 0.9$ µM, $K_2 = 1$ µM, $k_\mathrm{f} = 25$ h^{-1}, k=250 h^{-1}, $n = 2$, $m = 2$ and $p = 4$.

After cold treatment, calmodulin combines with Ca^{2+} and becomes activated according to a Hill function characterized by a Hill coefficient of n_0. The fraction of activated calmodulin, *CaM*, is thus given by eq. (3) where the cytosolic concentration $[\mathrm{Ca}^{2+}]$ is obtained by integration of eq. (1a) for a Ca^{2+}pulse, and by equating it to Z after integration of eqs. (2a)-(2b) in the case of Ca^{2+}oscillations:

$$CaM = \frac{[\mathrm{Ca}^{2+}]^{n_0}}{K_{\mathrm{Ca}}{}^{n_0} + [\mathrm{Ca}^{2+}]^{n_0}} \tag{3}$$

The peak of *CaM* shortly follows that of $[\mathrm{Ca}^{2+}]$. In this paper, we will consider that the pulsatile or oscillatory change in $[\mathrm{Ca}^{2+}]$ represents the effect of the cold stress and acts as signal input to the plant cold response pathway. The transient accumulation followed by an exponential decay of the Ca^{2+} concentration, with a half-decay time of the order of 15min, causes the downstream activation of the cold response pathway leading to the expression of CBF3 mRNA and the downstream expression of *COR* genes.

2. Kinetic equations for the cold stress response pathway

Based on the experimental data available in the literature (see Section 2 of the main text for details and references), we developed a model for the cold response pathway in plants, which is presented schematically in Figure 1. The time evolution of this model is governed by a system of 14 ordinary differential equations listed below as eqs. (4)-(17). In Section 4 below, the variables of the model are defined in Supplementary Table 1, while the definitions and numerical values of the parameters are given in Supplementary Table 2.

In writing these kinetic equations we take into account the following conservation relations for the total concentrations of the proteins CRLK1, CRPK1, MPK6, MPK4, HOS1, MYB15, and P1433:

$[\mathrm{CRLK1_t}] = [\mathrm{CRLK1}] + [\mathrm{CRLK1_a}]$
$[\mathrm{CRPK1_t}] = [\mathrm{CRPK1}] + [\mathrm{CRPK1_a}]$
$[\mathrm{MPK6_t}] = [\mathrm{MPK6}] + [\mathrm{MPK6P}]$
$[\mathrm{MPK4_t}] = [\mathrm{MPK4}] + [\mathrm{MPK4P}]$
$[\mathrm{HOS1_t}] = [\mathrm{HOS1}] + [\mathrm{HOS1_n}]$
$[\mathrm{MYB15_t}] = [\mathrm{MYB15}] + [\mathrm{MYB15P}]$
$[\mathrm{P1433_t}] = [\mathrm{P1433}] + [\mathrm{P1433P}]$.

It should be noted that there is no such conservation relation for the total concentrations of the other variables $[\mathrm{ICE1}]$, $[\mathrm{ICE1_P}]$, $[\mathrm{MCBF3}]$, $[\mathrm{CBF3}]$, $[\mathrm{CBF3_P}]$, $[\mathrm{MZAT12}]$, and $[\mathrm{ZAT12}]$ in the model because for these mRNA or protein species we consider both their synthesis and degradation, while for the other variables we only consider their interconversion between phosphorylated and unphosphorylated forms.

For the sake of clarity the differential equations are listed in five groups as follows:

(a) CRLK1 and CRPK1 are activated by CaM:

$$\frac{\mathrm{d[CRLK1_a]}}{\mathrm{d}t} = k_1\, CaM \cdot ([\mathrm{CRLK1_t}] - [\mathrm{CRLK1_a}]) - k_2[\mathrm{CRLK1_a}] \quad (4)$$

$$\frac{\mathrm{d[CRPK1_a]}}{\mathrm{d}t} = k_3 CaM \cdot ([\mathrm{CRPK1_t}] - [\mathrm{CRPK1_a}]) - k_4[\mathrm{CRPK1_a}] \quad (5)$$

In eqs. (4) and (5), the first term denotes the activation of CRLK1 or CRPK1 through interaction with calmodulin, while the second term relates to their inactivation.

(b) Phosphorylation of proteins MPK6P, MPK4P and HOS1 in the cytosol:

$$\frac{\mathrm{d[MPK6P]}}{\mathrm{d}t} = k_5[\mathrm{CRLK1_a}] \cdot \frac{[\mathrm{MPK6_t}]-[\mathrm{MPK6P}]}{K_1+[\mathrm{MPK6_t}]-[\mathrm{MPK6P}]} - v_1 \frac{[\mathrm{MPK6P}]}{K_2+[\mathrm{MPK6P}]} \quad (6)$$

$$\frac{\mathrm{d[MPK4P]}}{\mathrm{d}t} = k_6[\mathrm{CRLK1_a}] \cdot \frac{[\mathrm{MPK4_t}]-[\mathrm{MPK4P}]}{K_3+[\mathrm{MPK4_t}]-[\mathrm{MPK4P}]} - v_2 \frac{[\mathrm{MPK4P}]}{K_4+[\mathrm{MPK4P}]} \quad (7)$$

$$\frac{\mathrm{d[HOS1_n]}}{\mathrm{d}t} = k_7[\mathrm{CRLK1_a}] \cdot \frac{[\mathrm{HOS1_t}]-[\mathrm{HOS1_n}]}{K_5+[\mathrm{HOS1_t}]-[\mathrm{HOS1_n}]} - v_3 \frac{[\mathrm{HOS1_n}]}{K_6+[\mathrm{HOS1_n}]} \quad (8)$$

In eqs. (6), (7) and (8), the first term denotes the phosphorylation by active CRLK1 of MPK6, MPK4 or HOS1, while the second term relates to their dephosphorylation. All enzymatic terms are assumed to obey Michaelis-Menten kinetics.

(b) Activator ICE1 and inhibitors MYB15 and protein kinase 14-3-3:

$$\frac{\mathrm{d[MYB15]}}{\mathrm{d}t} = v_4 \frac{[\mathrm{MYB15_t}]-[\mathrm{MYB15}]}{K_7+[\mathrm{MYB15_t}]-[\mathrm{MYB15}]} - k_8[\mathrm{MPK6P}] \cdot \frac{[\mathrm{MYB15}]}{K_8+[\mathrm{MYB15}]} \quad (9)$$

$$\frac{\mathrm{d[ICE1]}}{\mathrm{d}t} = v_{\mathrm{s1}} - k_9[\mathrm{MPK6P}] \cdot \frac{[\mathrm{ICE1}]}{K_9+[\mathrm{ICE1}]} \cdot \frac{K_{\mathrm{I1}}}{K_{\mathrm{I1}}+[\mathrm{MPK4P}]} + v_5 \frac{[\mathrm{ICE1P}]}{K_{10}+[\mathrm{ICE1P}]} - v_{\mathrm{d1}} \frac{[\mathrm{ICE1}]}{K_{d1}+[\mathrm{ICE1}]} - k_{\mathrm{d1}}[\mathrm{ICE1}] \quad (10)$$

$$\frac{\mathrm{d[ICE1P]}}{\mathrm{d}t} = k_9[\mathrm{MPK6P}] \cdot \frac{[\mathrm{ICE1}]}{K_9+[\mathrm{ICE1}]} \cdot \frac{K_{\mathrm{I1}}}{K_{\mathrm{I1}}+[\mathrm{MPK4P}]} - v_5 \frac{[\mathrm{ICE1P}]}{K_{10}+[\mathrm{ICE1P}]} - v_{\mathrm{d2}} \frac{[\mathrm{ICE1P}]}{K_{\mathrm{d2}}+[\mathrm{ICE1P}]} \cdot \frac{[\mathrm{HOS1}_n]}{K_{a1}+[\mathrm{HOS1}_n]} - k_{\mathrm{d2}} \cdot [\mathrm{ICE1P}] \quad (11)$$

$$\frac{\mathrm{d[P1433P]}}{\mathrm{d}t} = k_{10}[\mathrm{CRPK1_a}] \cdot \frac{[\mathrm{P1433_t}]-[\mathrm{P1433P}]}{K_{11}+[\mathrm{P1433_t}]-[\mathrm{P1433P}]} - v_6 \frac{[\mathrm{P1433P}]}{K_{12}+[\mathrm{P1433P}]} \quad (12)$$

In eq. (9), the first term denotes the dephosphorylation of MYB15P, while the second term relates to the phosphorylation of MYB15 by MPK6P.

In eq. (10), the first term denotes the constant synthesis of ICE1, while the second term denotes the phosphorylation of ICE1 by MK6P, inhibited by MPK4P. The third term relates to the dephosphorylation of ICE1P; the fourth term represents the enzymatic degradation of ICE1, while the last term represents a linear, relatively small basal rate of degradation of ICE1 (this term allows the existence of a steady state in case parameter v_{d1} becomes too small).

In eq. (11), the first term denotes the increase in ICE1P though phosphorylation of ICE1 by MK6P, inhibited by MPK4P. The second term relates to the dephosphorylation of ICE1P; the third term represents the enzymatic degradation of ICE1P enhanced by $\mathrm{HOS1_n}$, while the last term represents a linear, relatively small basal rate of degradation of ICE1P (this term allows the existence of a steady state in case parameter v_{d2} becomes too small).

In eq. (12), the first term denotes the phosphorylation of P1433 by the active kinase CRPK1, while the second term relates to the dephosphorylation of P1433P.

(c) Output: CBF3 mRNA, phosphorylated and non-phosphorylated CBF3 protein:

$$\frac{\mathrm{d[MCBF3]}}{\mathrm{d}t} = v_{\mathrm{s2}} \frac{[\mathrm{ICE1P}]^n}{K_{\mathrm{a2}}{}^n+[\mathrm{ICE1P}]^n} \cdot \frac{K_{\mathrm{I2}}{}^m}{K_{\mathrm{I2}}{}^m+[\mathrm{MYB15}]^m} \cdot \frac{K_{\mathrm{I3}}{}^r}{K_{\mathrm{I3}}{}^r+[\mathrm{ZAT12}]^r} - v_{\mathrm{d3}} \frac{[\mathrm{MCBF3}]}{K_{\mathrm{d3}}+[\mathrm{MCBF3}]} - k_{\mathrm{d3}} \cdot [\mathrm{MCBF3}] \quad (13)$$

$$\frac{\mathrm{d[CBF3]}}{\mathrm{d}t} = k_{\mathrm{s1}} \cdot [\mathrm{MCBF3}] - k_{11}[\mathrm{P1433P}] \cdot \frac{[\mathrm{CBF3}]}{K_{13}+[\mathrm{CBF3}]} + v_7 \frac{[\mathrm{CBF3P}]}{K_{14}+[\mathrm{CBF3P}]} - v_{\mathrm{d4}} \frac{[\mathrm{CBF3}]}{K_{\mathrm{d4}}+[\mathrm{CBF3}]} - k_{\mathrm{d4}} \cdot [\mathrm{CBF3}] \quad (14)$$

$$\frac{\mathrm{d[CBF3P]}}{\mathrm{d}t} = k_{11}[\mathrm{P1433P}] \cdot \frac{[\mathrm{CBF3}]}{K_{13}+[\mathrm{CBF3}]} - v_7 \frac{[\mathrm{CBF3P}]}{K_{14}+[\mathrm{CBF3P}]} - v_{\mathrm{d5}} \frac{[\mathrm{CBF3P}]}{K_{\mathrm{d5}}+[\mathrm{CBF3P}]} - k_{\mathrm{d5}} \cdot [\mathrm{CBF3P}] \quad (15)$$

In eq. (13), the first term denotes the synthesis of *CBF3* mRNA (MCBF3) enhanced by ICE1P and inhibited by both MYB15 and ZAT12. The second term relates to the specific enzymatic degradation of MCBF3; the last term represents a linear, relatively small basal rate of

degradation of MCBF3 (this term allows the existence of a steady state in case parameter v_{d3} becomes too small).

In eq. (14), the first term denotes the synthesis of the CBF3 protein at a rate proportional to *CBF3* mRNA. The second term relates to the phosphorylation of CBF3 by the phosphorylated form of the kinase P1433; the third term represents the dephosphorylation of CBF3P; the fourth term relates to the specific enzymatic degradation of CBF3; the last term represents a linear, relatively small basal rate of degradation of CBF3 (this term allows the existence of a steady state in case parameter v_{d4} becomes too small).

In eq. (15), the first term relates to the phosphorylation of CBF3 by the phosphorylated form of the kinase P1433; the second term represents the dephosphorylation of CBF3P; the third term relates to the specific enzymatic degradation of CBF3P; the last term represents a linear, relatively small basal rate of degradation of CBF3P (this term allows the existence of a steady state in case parameter v_{d5} becomes too small).

(d) Negative feedback exerted by ZAT12 on CBF3 expression:

$$\frac{\mathrm{d[MZAT12]}}{\mathrm{d}t} = v_{\mathrm{s3}} \frac{[\mathrm{CBF3}]^s}{{K_{\mathrm{a3}}}^s + [\mathrm{CBF3}]^s} - v_{\mathrm{d6}} \frac{[\mathrm{MZAT12}]}{K_{\mathrm{d6}} + [\mathrm{MZAT12}]} - k_{\mathrm{d6}} \cdot [\mathrm{MZAT12}] \quad (16)$$

$$\frac{\mathrm{d[ZAT12]}}{\mathrm{d}t} = k_{\mathrm{s2}} \cdot [\mathrm{MZAT12}] - v_{\mathrm{d7}} \frac{[\mathrm{ZAT12}]}{K_{d7} + [\mathrm{ZAT12}]} - k_{\mathrm{d7}} \cdot [\mathrm{MZAT12}] \quad (17).$$

In eq. (16), the first term denotes the synthesis of *ZAT12* mRNA (MZAT12) induced by CBF3; the second term relates to the specific enzymatic degradation of MZAT12, while the last term represents a linear, relatively small basal rate of degradation of MZAT12 (this term allows the existence of a steady state in case parameter v_{d6} becomes too small).

In eq. (17), the first term denotes the synthesis of the ZAT12 protein at a rate proportional to the concentration of *ZAT12* mRNA (MZAT12); the second term relates to the specific enzymatic degradation of ZAT12, while the last term represents a linear, relatively small basal rate of degradation of ZAT12 (this term allows the existence of a steady state in case parameter v_{d7} becomes too small).

In eqs. (4)-(17), concentrations are defined with respect to the total cell volume. The subscripts *a*, *t* or *n* denote the activated form, the total amount and the nuclear form of the corresponding protein species, respectively.

3. Circadian gating

As recalled in the main text (see Section 4.7 for details and references), the cold induction of CBFs is gated by the circadian clock. The circadian clock components CCA1 and LHY positively regulate CBF transcription. In the morning hours, when CCA1 and LHY protein levels peak, the transcript levels of CBFs also peak; in the evening hours, when CCA1 and LHY protein levels are low, transcript levels for CBFs are low. The transcript levels for CBFs thus oscillate, with peaks and troughs occurring at about ZT8 and ZT20, respectively. To incorporate the circadian gating into the transcription of CBF3, we multiply the maximal synthesis of CBF3 mRNA v_{s2} by a Hill function g, defined as

$$g = \frac{C^h}{K_C^h + C^h} \qquad (18)$$

where a variable C (which could represent the level of a circadian clock protein such as CCA1 or LHY) oscillates in a circadian manner according to the phenomenological equation (19):

$$C = C_0 + C_1(1 + \sin(2\pi(t-2)/p_c))^q \qquad (19)$$

We take the values of C_0, C_1, q, K_c, h, and p_c as equal to 0.01, 1, 4, 0.15, 4, and 24, respectively.

The kinetic equation (13) for CBF3 transcription is now replaced by equation (20):

$$\frac{\mathrm{d[MCBF3]}}{\mathrm{d}t} = v_{\mathrm{s2}} . g . \frac{[\mathrm{ICE1P}]^n}{K_{\mathrm{a2}}{}^n + [\mathrm{ICE1P}]^n} \cdot \frac{K_{\mathrm{I2}}{}^m}{K_{\mathrm{I2}}{}^m + [\mathrm{MYB15}]^m} \cdot \frac{K_{\mathrm{I3}}{}^r}{K_{\mathrm{I3}}{}^r + [\mathrm{ZAT12}]^r} - v_{\mathrm{d3}} \frac{[\mathrm{MCBF3}]}{K_{\mathrm{d5}} + [\mathrm{MCBF3}]} - k_{\mathrm{d3}} \cdot [\mathrm{MCBF3}] \qquad (20).$$

4. Model parameterization and sensitivity analysis

4.1 Model parameterization

Not counting the fraction of activated calmodulin *CaM*, the kinetic equations (4)-(17) which govern the time evolution of the model for the cold response pathway involve 77 parameters, which are listed in Table 2 below. Most of these parameter values were not (yet) characterized experimentally. For example, kinetic studies have yet to be performed to yield values for the

various rates of phosphorylation or dephosphorylation, for the Michaelis constants that characterize these enzyme reactions, or for the activation or inhibition constants that measure the regulatory interactions. A similar remark holds for other parameter values which have not yet been determined, such as the total concentrations of the different molecular species involved in the pathway. For all these parameters we made a semi-arbitrary choice, in a physiologically reasonable range. Selecting parameter values in a semi-arbitrary manner means, for example, that the different activation or inhibition constants, were assigned values of the order of the concentrations of the various molecular species involved in these regulations in the model, and not smaller or larger by two or more orders of magnitude than these concentrations, because in the latter case the corresponding regulations would remain ineffective. A similar remark holds for the choice of Michaelis constants. For the brief initial Ca^{++} pulse, we chose values for the kinetic parameters ensuring that the pulse is over in minutes, as observed in the experiments (Plieth et al. 1999). For Ca^{2+} oscillations, we selected parameter values in eqs. (2a)-(2d) as described in Section 1.2 of Supplementary Material, so as to obtain a period of the order of 150 s as observed in plant guard cells (Allen et al., 2001).

The choice of parameter values was guided by the constraint of matching the observed characteristics of the *CBF3* expression, which is the major response measured in the experiments. This was achieved by trial and error, i.e. by performing simulations for a large set of parameter values and selecting a set that produced a *CBF3* mRNA peak in a range extending from 1 h to 6 h after the cold stress, as observed in different experiments. Thus, *CBF3* mRNA displayed a peak at 1 h (Medina et al., 2011), 3 h (Zarka et al., 2003; Agarwal et al., 2006), 4 h (Vogel et al., 2005), and 6 h (Chinnusamy et al., 2003) after the cold stress. Although detailed data for the time course of *CBF3* mRNA remain scarce, we may collect information about the amplitude, peak time, and half-width of *CBF3*. The amplitudes of *CBF3* mRNA is defined as the maximum value after cold stress compared to the steady state level prior to the stress. The lower and upper boundaries define a range of about 20 – 500 for the fold-change in amplitude (Dong et al., 2011; Chinnusamy et al., 2003). A smaller amplitude change of the order of 10 may obtain when decreasing the temperature from 20°C to ~10°C instead of 4°C (Zarka et al., 2003). The upper value is observed in the *ice1* mutant, which gives a variation of about 460 fold with respect to WT.

In regard to the half-width of the peak in *CBF3* mRNA, as the time points in experimental data are sparse, it is not easy to find two timepoints corresponding to about 50% of the maximum of the peak in *CBF3* mRNA. The experimental points are often distant from the half-maximum level. Therefore, we can only infer a reasonable range for the half-width. In the experiments, 12h after cold signaling the *CBF3* mRNA level is very low and already close to the steady-state level. The peak level is observed between 1h and 6h after the cold stress. This gives an upper value of some 6h for the half-width, assuming that *CBF3* expression rises up to, and thereafter decreases down to, 50% of the peak level, at 3h and 9h after the cold stress,

respectively. The typical peaking time at 3h gives a lower boundary for the half-width of about 3h in a symmetric peak hypothesis. We therefore considered a range of 3h-6h for the half-width of the peak in *CBF3* expression induced by the cold signal.

The degradation rate of *CBF3* mRNA was selected on the basis of its half-life measured at low and ambient temperatures. At low temperature, the *CBF3* mRNA is more stable and has consequently a longer half-life. Experiments showed that the *CBF3* transcript has a half-life of 7.5 min at warm temperatures, which gives an approximately degradation rate of *CBF3* mRNA at 20°C of the order of 5.5 nM/h, while at low temperature this degradation rate is of the order of 0.55 nM/h (Zarka et al., 2003).

After using the selected parameter values to compare in Figures 2 and 3 the predictions of the model with experimental observations on the WT, we compared in Figure 5 and in Supplementary Figures S2 and S3 the theoretical predictions with experimental observations in the mutants *ice1* and *myb15*, or in conditions of overexpression of HOS1 (HOS1-OX) and of ZAT12 (ZAT12-OX). This was done respectively by reducing or increasing the rates of expression of the corresponding genes with respect to the basal values listed in Table 2 for these parameters, as described in the legends of the figures.

4.2 Sensitivity analysis

Due to the lack of quantitative information on the values of most parameters of the model, an optimization approach appears unfeasible. However, as an alternative and to obtain insight into the effect of changing the basal parameter values listed in Table 2, we performed a sensitivity analysis so as to determine those parameters toward which the system is most sensitive. The sensitivity analysis was performed with respect to three properties of the peak of *CBF3* expression elicited by a cold stress: the amplitude of the peak in *CBF3* mRNA (Suppl. Figure S5A), the timing of this peak (Suppl. Figure S5B), and the half-width, measuring the duration of the response (Suppl. Figure S5C). Each parameter is varied one at a time from 10^{-3} to 10^{3} times its basal value listed in Table 2, while holding the other parameters fixed at their values given in this Table (the Hill coefficients remain fixed at their values listed in Table 2). For each of the parameters we determine the range (denoted by the vertical bar) in which the parameter value satisfies the constraint prescribed on one of the three properties of the cold-induced peak in CBF3 expression, on the basis of the observations described above in Supplementary Material Section 4.1. The three constraints are addressed in turn, independently. Parameter sensitivity is measured by determining numerically the range of values satisfying the particular constraint and dividing then the upper bound by the lower bound of this range. The smaller the range and the ratio for any given parameter, the larger the sensitivity of the system towards this parameter. In Supplementary Figure S5 we provide a

graphical representation of these results and show, for each parameter, the range satisfying one of the three constraints retained for sensitivity analysis.

In Suppl. Figure S5A the vertical bar displays for any given parameter the range in which the maximum of the peak is between 20 and 500 times larger than the steady-state level of *CBF3* mRNA prior to the cold stress. For the amplitude of the *CBF3* mRNA peak, parameter k_5, which measures the rate constant for phosphorylation of MPK6 by the activated CRLK1 protein kinase, appears to be the most sensitive; increasing the value of this parameter above 6.78 or decreasing it below 3.19 with respect to its basal value of 5.80 produces a peak that fails to obey the constraint on the amplitude of the peak in *CBF3* mRNA. The ratio of 2.13 of these two boundary values yields a measure of the sensitivity with respect to this parameter. Other parameters appear to be nearly as sensitive, while some can be varied over several orders of magnitude without failing to obey the constraint on the amplitude of the *CBF3* mRNA peak. The 30 most sensitive parameters determined for this constraint are listed in Table 3 below, in the order of decreasing sensitivity; also listed are the boundaries of the range in which the constraint is satisfied. Supplementary Figure 5A and Table 3 indicate that the most sensitive parameters with respect to the amplitude of the *CBF3* expression peak are those that relate to steps which immediately follow the activation of calmodulin after the cold stress, namely, activation of calmodulin by Ca^{2+}, activation of CRLK1 and MPK6, and the parameters related to MYB15 and ICE1 which, respectively, inhibit and enhance the synthesis of *CBF3* mRNA.

In Suppl. Figure 5B the vertical bar indicates for each parameter the range in which the peak in in *CBF3* mRNA occurs between 1h and 6 h after the cold stress (see Supplementary Material, Section 4.1). As indicated in Table 4 which lists the 25 most sensitive parameters that most impinge on the timing of the *CBF3* mean peak, the ratios of parameter values that define the range satisfying the constraint extend from ~ 10 to > 10^6. The other non-listed parameters all have ratios > 10^6. Among the most sensitive parameters are the total concentration of MYB15, which protein directly controls the level of CBF3 mRNA through inhibition of *CBF3* expression (see scheme of the model in Figure 1), and the rates of degradation of *CBF3* mRNA. Beyond the differences between the three properties of the peak in *CBF3* expression, the comparison of the panels in Supplementary Figure S5 suggests that the timing of the peak in *CBF3* mRNA represents a more robust property than its amplitude or half-width, given that the ranges of parameter values satisfying the constraint on the timing of the peak in (B) are more extended than in (A) and (C).

Finally, in Suppl. Figure 5C the vertical bar indicates for each parameter the range in which the parameter value yields a cold-induced peak in in *CBF3* mRNA with a half-width comprised between 3h and 6h. The half-width is the difference between the times at which *CBF3* mRNA after a cold stress rises up to 50% and subsequently decreases down to 50% of its maximum.

Listed in Table 5 are the 30 most sensitive parameters determined with respect to the half-width of the peak in *CBF3* expression following a cold stress. As noted above, the ranges are narrower than for the timing of the peak. Among the parameters most sensitive for this characteristic of the peak are the levels of CRLK1 and MPK6, located upstream in the cold response pathway, and kinetic parameters such as the rate constants measuring phosphorylation and dephosphorylation of MYB15 (which protein controls the expression of *CBF3*), and the constants measuring calmodulin activation. The latter result holds with the view that the amplitude of the initial activation of calmodulin by Ca^{2+} following a drop in temperature governs the duration of the peak in *CBF3* expression.

Some parameters are the most sensitive with respect to more than one characteristic property of the response. Thus, the kinetic constant k_8, which measures the rate of phosphorylation of MYB15 by MPK6, appears to tightly control the amplitude and half-width of the CBF3 peak, but not so much its timing. Relative to other parameters, some possess only a narrow range in which they satisfy all three properties considered in the sensitivity analysis. This is the case for k_1 and v_{d2} which measure, respectively, the rate of activation of calmodulin by CRLK1, and the rate of degradation of phosphorylated ICE1.

For the sensitivity analysis illustrated by Fig. S5, we held the ratio (v_{d3W}/v_{d3C}) equal to 10, in agreement with the experimental observation that the rate of CBF3 mRNA degradation decreases by one order of magnitude at 4°C (see section 4.1 above). Moreover, to compare the sensitivities towards the different parameters, the peak of *CBF3* expression elicited by the cold stress was determined for the same set of initial conditions, namely, the steady state corresponding to the basal set of parameter values, with $v_{d3} = v_{d3W}$ and (v_{d3W}/v_{d3C})=10. The ranges characterizing the sensitivities towards the various parameters, as determined in Fig. S5 and Tables 3-5, depend, however, on the choice of initial conditions. To illustrate this dependence, we determine through numerical integration of the kinetic equations, for each parameter value, the steady state that corresponds to the value of the parameter that is changed from 0.001 to 1000 times its basal value, while all other parameters are kept at their basal value, with $v_{d3} = v_{d3W}$ (warm conditions, prior to the cold stress). Our goal was to take this steady-state as initial condition for determining the characteristics of the *CBF3* expression peak induced by the cold stress. In trying to do so we encountered, however, a difficulty. For some parameters indeed, a change from the basal value induces sustained oscillations in *CBF3* expression, as reported in Section 4.6 in the main text. In these conditions, it is impossible to determine a stable steady state from which the system would start to generate a single pulse of *CBF3* expression after the cold stress is applied.

The occurrence of oscillations is illustrated in Fig. S6 for the case of parameter K_{Ca}, which measures the threshold for half-maximum activation of calmodulin by Ca^{2+}. In Fig. S6A the initial conditions correspond to the steady state determined for $v_{d3} = v_{d3W}$ and for the basal set

of parameter values, except for the parameter that is changed, for which we take the modified value (i.e., 0.2 multiplied by 0.6, 0.55, 0.5, or 0.001). In Fig. S6B the initial conditions correspond to the steady state determined for $v_{d3} = v_{d3W}$ when all parameter values are kept at their basal values. When the basal value of K_{Ca}, 0.2, is multiplied by 0.6, both in (A) and (B) the cold stress induces a transient peak of *CBF3* expression before the cold response pathway reaches a stable steady state. In contrast, when the basal value is multiplied by 0.5 or 0.01, sustained oscillations in *CBF3* mRNA develop, while damped oscillations are found when the basal value is multiplied by 0.55. Not all parameters behave in this way. For many parameters, the change from 0.001 to 1000 times the basal parameter value does not induce oscillations, so that a stable steady state is reached when $v_{d3}=v_{d3W}$ in warm conditions, before the cold stress is applied.

We selected five parameters for which no oscillations were induced by the cold stress, to determine the effect of the initial conditions on the sensitivity analysis. We show in Fig. S7 the results of a comparative analysis of sensitivity for the parameters K_{Ca}, k_1, k_2, K_1, $CRLK1_t$. The data in the left panels were obtained upon taking as initial condition the steady state corresponding to the basal set of values for all parameters in warm conditions, while the data in the right panels pertain to initial conditions that correspond to the steady state obtained similarly, but with the actual value of the sole parameter that is being changed. The comparison of the left and right panels indicates that the range in which the three constraints on the peak of *CBF3* expression are satisfied depends on the initial conditions. We can conclude from this figure that, at least for the 5 parameters considered, the parameter range satisfying each of the 3 constraints on the *CBF3* mRNA peak is slightly larger for the initial conditions considered in Fig. S7D-F compared to those in Fig. S7A-C.

The interest of sensitivity analysis is to allow us to determine which parameters might be modified experimentally to affect most effectively the amplitude, timing, or half-width of the peak in *CBF3* expression elicited by the cold stress.

5. Supplementary Figures and Tables

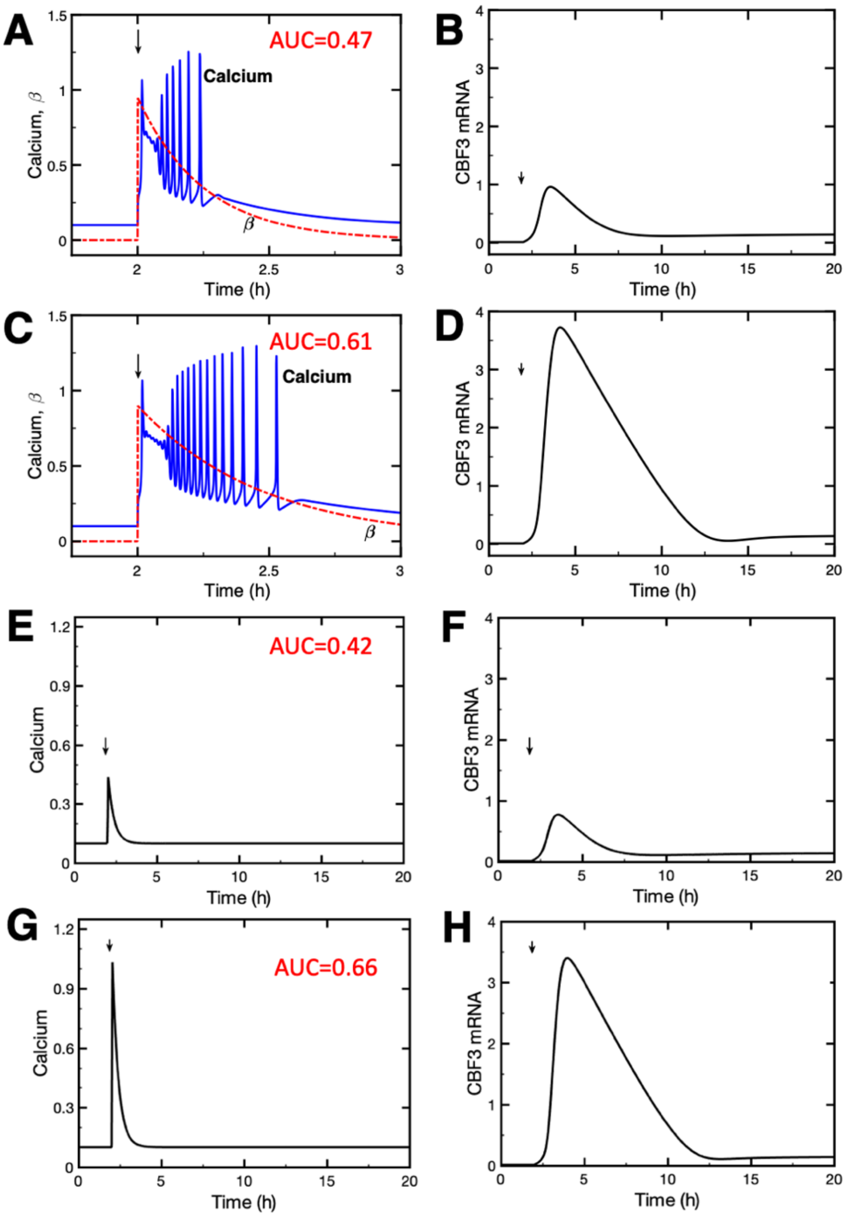

Figure S1. The amounts of Ca^{2+} and corresponding CBF3 mRNA levels after cold stress: Comparison of Ca^{2+} oscillations with a single Ca^{2+} pulse. The increase in Ca^{2+} triggered by cold signaling is determined by measuring the area under curve (AUC) for the time course of Ca^{2+} after the cold stress applied at t=2 h. When the AUCs are 0.47 in the course of Ca^{2+} oscillations (A) and 0.42 in a single Ca^{2+} pulse (E), the corresponding maximum values of *CBF3* mRNA are around 1.0, as shown in (B) and (F), respectively. When AUCs are about 0.61 in the course of Ca^{2+} oscillations (C) and 0.66 for a single Ca^{2+} pulse (G), the

corresponding maximum values of *CBF3* mRNA are around 3.5, as shown in (D) and (H), respectively. The AUCs are computed by the numerical summation of area under the Ca^{2+} curve for time > 2 h with step size equal to 0.001. Parameter values for A-D are as in Figure 4; for E-H parameter values are as in Figure 2, except for E, F where I_{INmax}=10 and $\Theta_{\mathrm{drop}} = 0.035$.

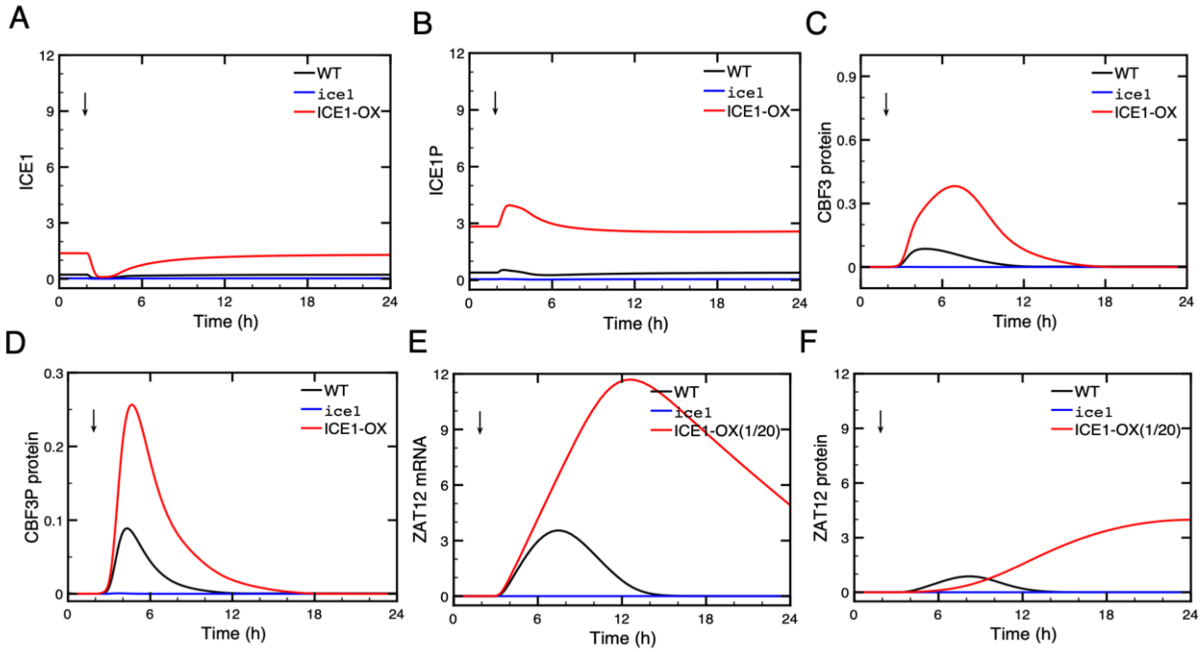

Figure S2. Time evolution for *ice1* mutant (red curves) and overexpression OX-ICE1 (black curves) in response to cold shock at 2h. For *ice1* mutant and OX-ICE1, the synthesis rate of ICE1, v_{s1}, is equal (in nM/h) to 0.036 and 0.36, respectively. The panels show the time course of the levels of ICE1 (A), ICE1P (B), CBF3 protein (C), CBF3P protein (D), ZAT12 mRNA (E) and ZAT12 protein (F). In (E) and (F) some levels are divided by 20 for the overexpression case. Except for v_{s1}, parameter values are listed in Supplementary Table 2.

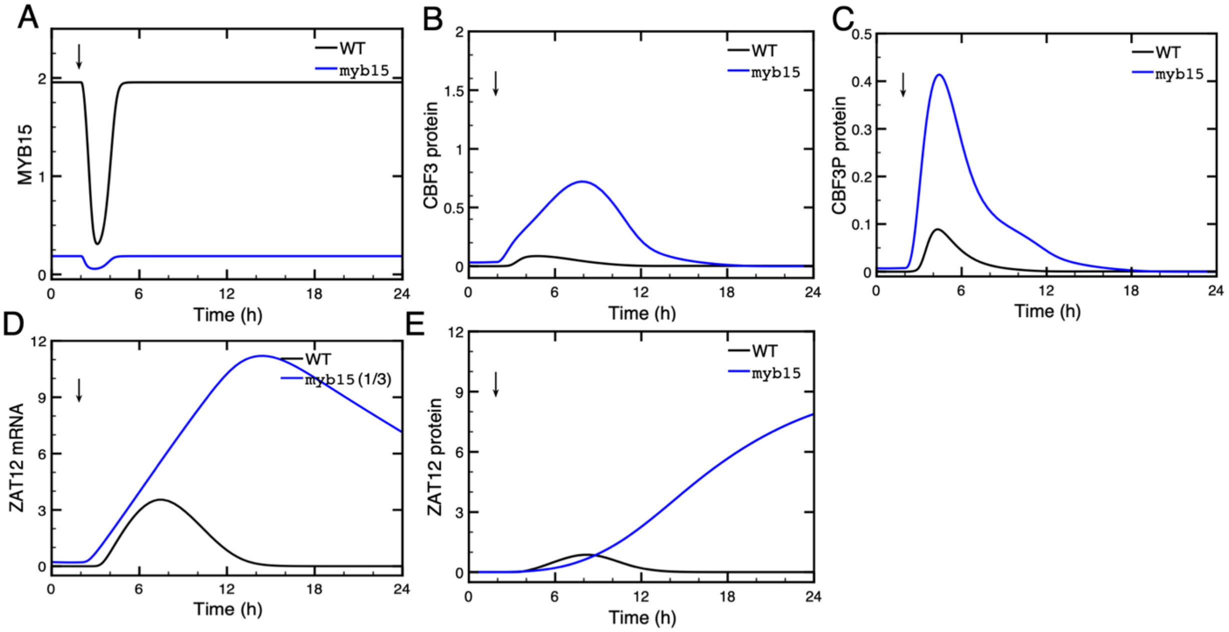

Figure S3. Time evolution for wild type WT and *myb15* mutant in response to cold shock at 2h. The black and blue curves pertain to the WT and *myb15* mutant, respectively. For the *myb15* mutant, the total amount of MYB15 is 0.2 nM, tenfold lower than in WT. The curves show the time course of MYB15 protein (A), CBF3 protein (B), CBF3P protein (C), *ZAT12* mRNA(D) and ZAT2 protein (E) in wild type and *myb15* mutant. Parameter values are listed in Supplementary Table 2. The time evolution of *CBF3* mRNA in response to a cold stress in WT and in the *myb15* mutant is shown in Fig. 5C in main text

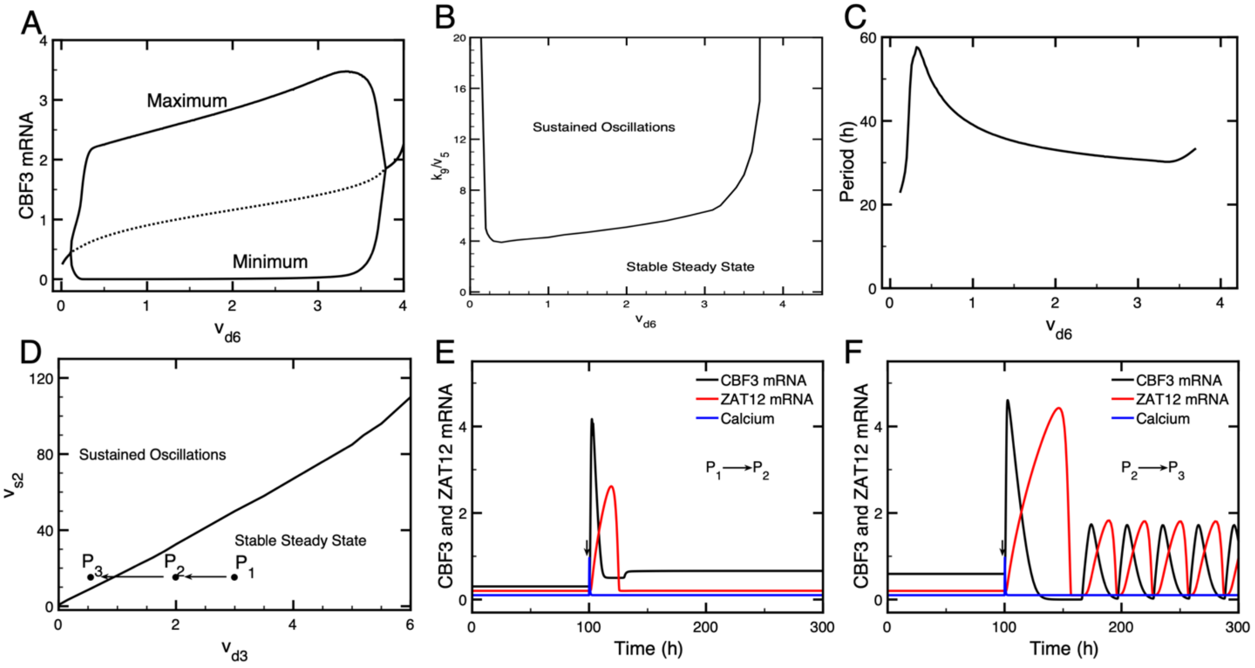

Figure S4. Domain of ZAT12-induced oscillations in CBF3 expression. Bifurcation analysis of eqs. (4)-(17) for the cold response pathway is performed by means of the XPP software (http://www.math.pitt.edu/~bard/xpp/xpp.html). (A) Bifurcation diagram showing the envelope of CBF3 mRNA oscillations as a function of the ZAT12 mRNA degradation rate v_{d6}. The concentration of CBF3 mRNA reaches a stable steady state when v_{d6} (in nM/h) is less than 0.1 and above a value between 3.75 and 3.8. When the value of v_{d6} is greater than 0.1 and less than 3.75, the steady state is unstable (dotted line) and the concentration of CBF3 mRNA oscillates (see Figure 6, main text). Shown are the maximum and minimum values of CBF3 mRNA in the course of sustained oscillations (solid lines). (B) Domain of sustained oscillations as a function of ratio k_9/v_5 versus v_{d6}. Sustained oscillations occur when the ZAT12 degradation rate v_{d6} ranges from 0.1 to 3.75, and the ratio of rate constants for ICE1 phosphorylation, k_9, and dephosphorylation, v_5, ranges from about 3.9 to infinity. When the ratio k_9/v_5 is less than 3.9, the system evolves to a stable steady state for all v_{d6} values. If v_{d6} is less than 0.1, the system reaches a stable steady state for all k_9/v_5 values. (C) Period of CBF3 oscillations as a function of the ZAT12 mRNA degradation rate v_{d6}. (D) Domain of sustained oscillations as a function of v_{s2} and v_{d3}, which respectively measure the rates of synthesis and degradation of CBF3 mRNA. (E) Time evolution of CBF3 mRNA as the system goes from point P_1, corresponding to a stable steady state in (E), to point P_2, which also corresponds to a stable steady state. (F) Time evolution of CBF3 mRNA as the system goes from the stable point P_1 to point P_3 located in the domain of sustained oscillations in (E). The value of parameter v_{d3} for points P_1, P_2, P_3 is equal (in nM/h) to 3.0, 2.0 and 0.55 respectively. Parameter values are listed in Supplementary Table 2 except for those listed in legend of Figure 6.

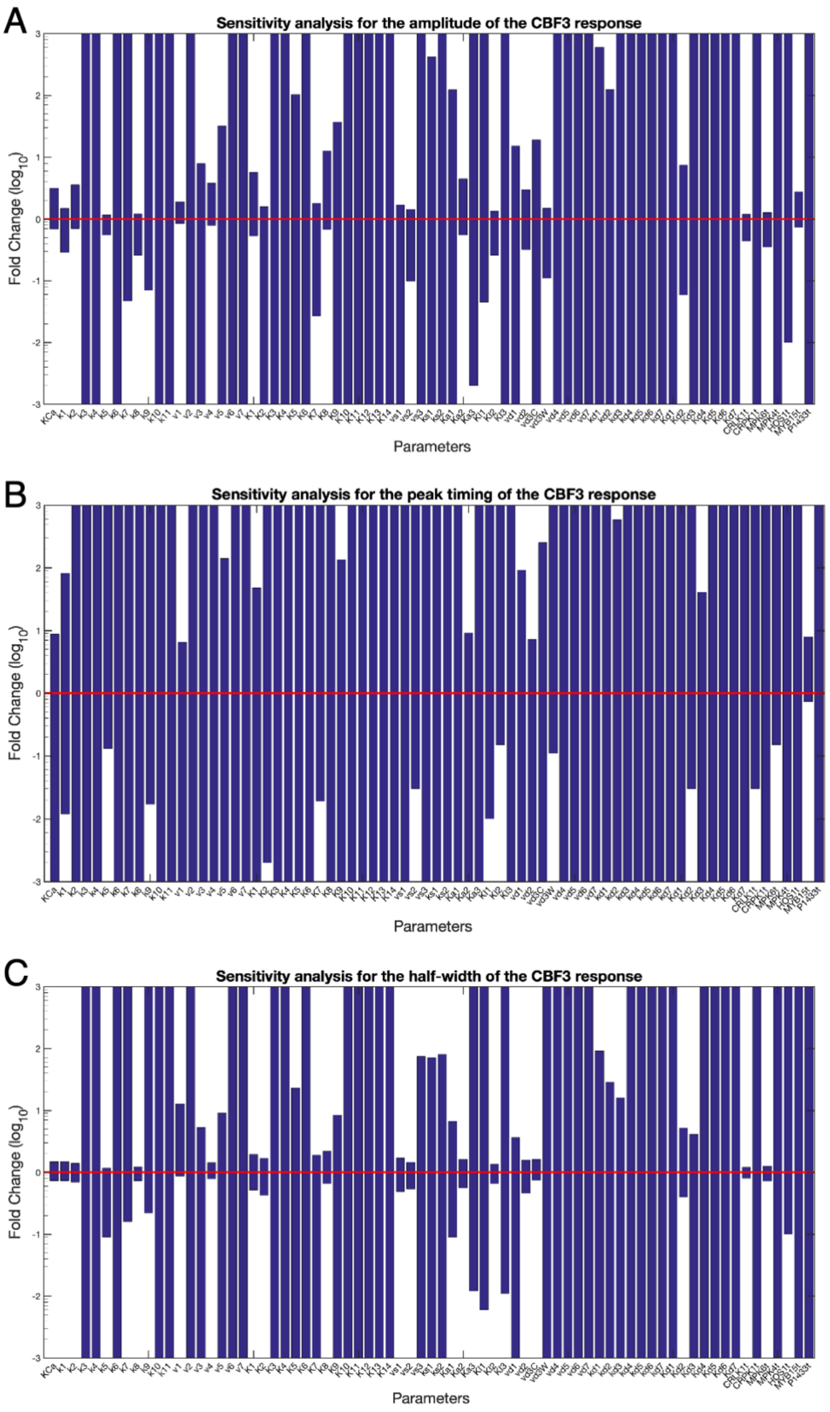

Figure S5. Sensitivity analysis for the amplitude (A), timing (B) and half-width (C) of the peak in *CBF3* mRNA elicited by a cold stress. In each panel we vary one parameter at a time in a range going from 10^{-3} to 10^{3} times the basal value listed in Table 2, while holding other parameters at their basal values listed in this Table. The name of each of the 73 parameters is indicated on the horizontal axis (reading the names may require enlarging the figure). The steady state determined upon assigning to all parameters their basal values listed in Table 2 is

taken as initial condition for determining the three characteristics of the peak in CBF3 expression elicited by the cold stress. The bar on top of a given parameter indicates the range (in log scale) in which the constraints on one of these three characteristics are satisfied. In (A) the range corresponds to values of the parameter for which the amplitude of the peak in *CBF3* mRNA is 20 times to 500 times larger than the steady-state level observed prior to the drop in temperature. In (B) the range corresponds to values of the parameter for which the peak in *CBF3* mRNA occurs between 1h and 6h after the cold stress. In (C) the range corresponds to values of the parameter for which the half-width of the peak in *CBF3* mRNA elicited by the cold stress extends from 3h to 6h. To determine the half-width we determine the difference between the times at which the peak rises up to 50% of its maximum and subsequently decreases down to 50% of the maximum. The choice of the three constraints is based on experimental observations, as explained in Section 4.1 of Supplementary Material as well as in the legends to Supplementary Tables 3-5.

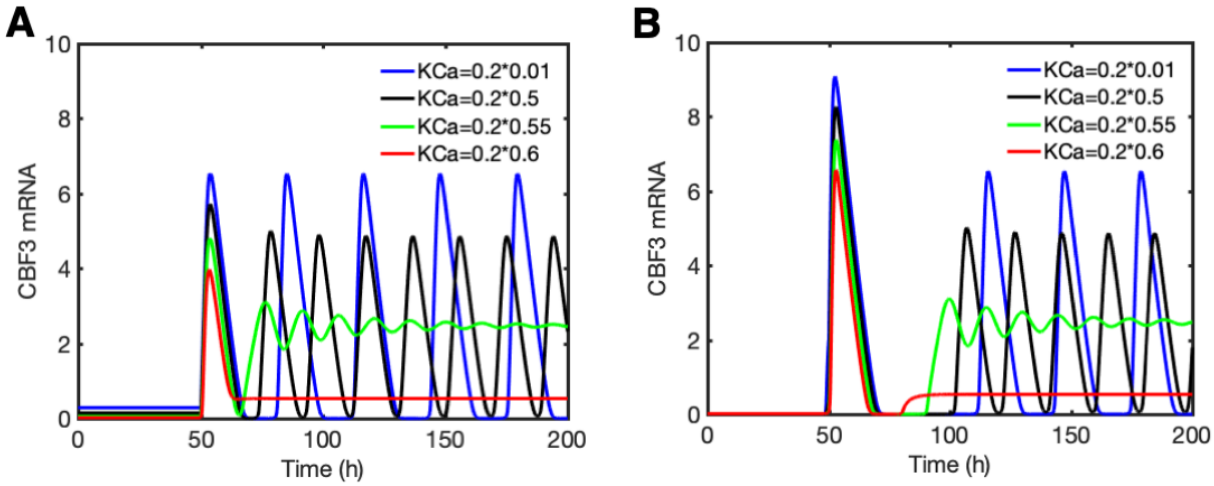

Figure S6. *CBF3* expression elicited by a cold stress, without or with oscillations. For some parameters, such as K_{Ca}, the cold stress elicits a single peak or oscillations of *CBF3* mRNA. Here, oscillations are induced at smaller values K_{Ca} after the cold signal (with v_{d3} = v_{d3C}) , when K_{Ca} is decreased below its basal value equal to 0.2. When the basal value of K_{Ca} is multiplied by 0.6, 0.55, 0.5, or 0.01, the evolution of *CBF3* mRNA displays a single peak (red curve), damped oscillation (green curve), or sustained oscillations (black and blue curves), respectively. Other (basal) parameter values are listed in Supplementary Table 2. The initial conditions in (A) correspond to the steady state determined for each value of K_{Ca} while all other parameters are their basal values, in warm conditions (v_{d3}=v_{d3W}). The initial values in (B) correspond to the steady state determined similarly, when all parameters are their basal values.

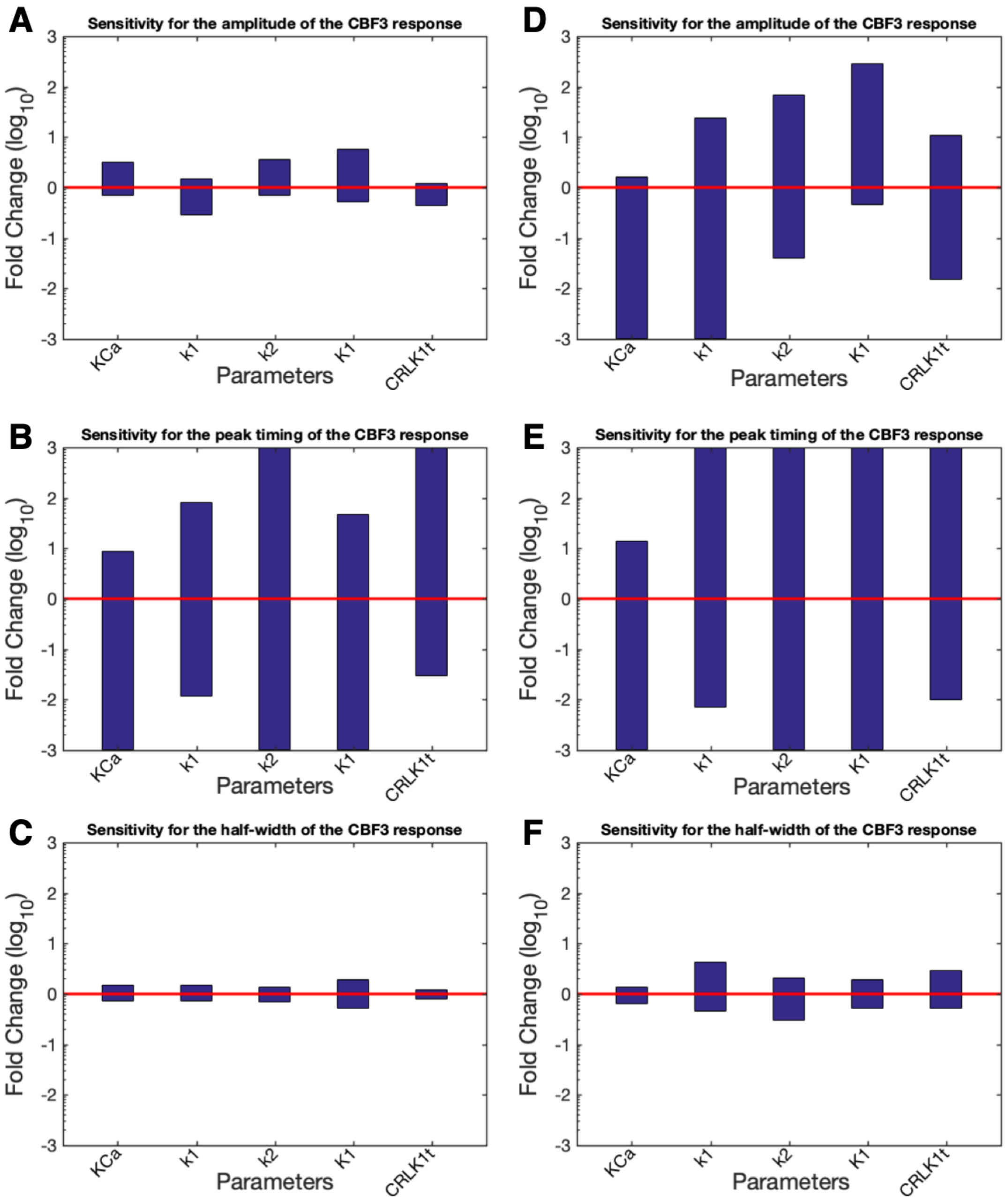

Figure S7. Parameter sensitivity : Dependence on initial conditions. To perform a comparative sensitivity analysis we consider two sets of initial conditions for which we determine, as in Fig. S5, the range of variation in which each of 5 selected parameters obeys one of the three constraints on the peak of *CBF3* expression defined in the legend to Fig. S5 and in Section 4.2 of Supplementary Material. For these parameters, K_{Ca}, k_1, k_2, K_1, and CRLK1_t, no oscillations occur in warm conditions ($v_{d3}=v_{d3W}$) when the parameter is varied, and a steady state is reached, as illustrated in Fig. S6 for K_{Ca}. The peak in *CBF3* expression is triggered by the shift from warm to cold conditions; this cold stress is accompanied by a Ca^{2+} pulse and by a change of v_{d3} from the value v_{d3W} to v_{d3C}. Sensitivity is determined in (A) and (D) with respect to the amplitude of the peak, in (B) and (E) with respect to its timing, and in

C) and (F) in regard to the half-width. The data in panels A, B and C are replotted from Fig. S5A, B, C, respectively. In the left panels (A)-(C) the initial conditions correspond to the steady state reached when all parameters have their basal values listed in Table 2, with $v_{d3} = v_{d3W}$. In the right panels the initial conditions correspond to the steady state obtained similarly, for the basal set of parameter values, except for the parameter that is changed (i.e., K_{Ca}, k_1, k_2, K_1, or $CRLK1_t$), for which the modified value is taken in a range extending from 0.001 to 1000 times the basal value.

Supplementary Table 1. Variables of the model for the plant cold stress response pathway

Symbol	Definition
CRLK1a	Activated CRLK1 protein
CRPK1a	Activated CRPK1 protein
MPK6P	Phosphorylated MPK6 protein
MPK4P	Phosphorylated MPK4 protein
HOS1n	Nuclear HOS1 protein
MYB15	Unphosphorylated MYB15 protein
ICE1	Unphosphorylated ICE1 protein
ICE1P	Phosphorylated ICE1 protein
P1433P	Phosphorylated 14-3-3 protein
MCBF3	CBF3 mRNA
CBF3	Unphosphorylated CBF3 protein
CBF3P	Phosphorylated CBF3 protein
MZAT12	ZAT12 mRNA
ZAT12	ZAT12 protein

Supplementary Table 2. Parameter definitions and numerical values

Listed are the parameters that appear in eqs. (4)-(17), which govern the time evolution of the model for the pathway underlying the plant response to cold stress, schematized in Figure 1. Also given are the values of these parameters used for numerical simulations of the model in Figure 2. These parameter values have been selected semi-arbitrarily so as to yield the order of magnitude of the time course observed experimentally for CBF3 expression following a cold stress. In the absence of quantitative information on the kinetic parameters and on the intracellular levels of mRNA and protein species, the concentration scale (in nM) should be considered as tentative.

Parameter	Description	Value	Unit
CaM	Fraction of activated calmodulin, determined as a function of [Ca^{2+}] according to eq. (3)		
n_0	Hill coefficient for calmodulin activation by Ca^{2+}	4	
K_{Ca}	Threshold for half-maximum activation of calmodulin by Ca^{2+}	0.2	nM
k_1	Apparent first-order rate constant for activation of CRLK1 by calmodulin	6.5	h^{-1}
k_2	First-order rate constant for inactivation of activated CRLK1 protein	6.15	h^{-1}
k_3	Apparent first-order rate constant for activation of CRPK1 by calmodulin	4.5	h^{-1}
k_4	First-order rate constant for inactivation of activated CRLK1 protein	4.2	h^{-1}
k_5	Rate constant for phosphorylation of MPK6 by activated CRLK1 protein	5.8	h^{-1}
k_6	Rate constant for phosphorylation of MPK4 by activated CRLK1 protein	4.8	h^{-1}
k_7	Rate constant for transport of cytosolic HOS1 into the nucleus through putative phosphorylation by activated CRLK1 protein	6.15	h^{-1}
k_8	Rate constant for phosphorylation of MYB15 by MPK6 protein	4.8	h^{-1}
k_9	Rate constant for phosphorylation of ICE1 by phosphorylated MPK6 protein	15	h^{-1}
k_{10}	Rate constant for phosphorylation of P1433 by activated CRPK1 protein	2.2	h^{-1}
k_{11}	Rate constant for the phosphorylation of CBF3 protein by phosphorylated P1433 protein	3	h^{-1}
v_1	Rate of dephosphorylation of phosphorylated MPK6 protein	5.45	nM/h
v_2	Rate of dephosphorylation of phosphorylated MPK4 protein	4.45	nM/h
v_3	Rate of transport of HOS1 out of the nucleus through putative dephosphorylation	1.95	nM/h
v_4	Rate of dephosphorylation of phosphorylated MYB15 protein	4.45	nM/h

v_5	Rate of dephosphorylation of phosphorylated ICE1 protein	0.8	nM/h
v_6	Rate of dephosphorylation of phosphorylated P1433 protein	1.05	nM/h
v_7	Rate of dephosphorylation of phosphorylated CBF3 protein	1	nM/h
K_1	Michaelis constant for phosphorylation of MPK6 by activated CRLK1 protein	0.5	nM
K_2	Michaelis constant for dephosphorylation of phosphorylated MPK6 protein	0.5	nM
K_3	Michaelis constant for phosphorylation of MPK4 by activated CRLK1 protein	0.5	nM
K_4	Michaelis constant for dephosphorylation of phosphorylated MPK4 protein	0.5	nM
K_5	Michaelis constant for phosphorylation of HOS1 by activated CRLK1 protein	0.5	nM
K_6	Michaelis constant for dephosphorylation of phosphorylated HOS1 protein	0.5	nM
K_7	Michaelis constant for dephosphorylation of phosphorylated MYB15 protein	0.5	nM
K_8	Michaelis constant for phosphorylation of MYB15 by phosphorylated MPK6 protein	0.5	nM
K_9	Michaelis constant for phosphorylation of ICE1 by phosphorylated MPK6 protein	0.45	nM
K_{10}	Michaelis constant for dephosphorylation of phosphorylated ICE1 protein	0.8	nM
K_{11}	Michaelis constant for phosphorylation of P1433 by activated CRPK1 protein	0.65	nM
K_{12}	Michaelis constant for dephosphorylation of phosphorylated P1433 protein	0.65	nM
K_{13}	Michaelis constant for phosphorylation of CBF3 by phosphorylated P1433 protein	0.6	nM
K_{14}	Michaelis constant for dephosphorylation of phosphorylated CBF3 protein	0.58	nM
v_{s1}	Rate of synthesis of ICE1 protein	0.18	nM/h
v_{s2}	Maximum rate of synthesis of CBF3 mRNA	18	nM/h
v_{s3}	Maximum rate of synthesis of ZAT12 mRNA	2.2	nM/h
k_{s1}	Apparent first-order rate constant for the synthesis of CBF3 protein, assumed to be proportional to CBF3 mRNA	0.25	h^{-1}
k_{s2}	Apparent first-order rate constant for the synthesis of ZAT12 protein, assumed to be proportional to ZAT12 mRNA	0.5	h^{-1}
K_{a1}	Michaelis constant for activation by nuclear HOS1 of phosphorylated ICE1 protein degradation	0.5	nM
K_{a2}	Threshold constant for activation of *CBF3* expression by phosphorylated ICE1 protein	0.45	nM
K_{a3}	Threshold constant for activation of $ZAT12$ expression by CBF3 protein	0.1	nM

K_{I1}	Threshold constant for inhibition of ICE1 by phosphorylated MPK4 protein	0.45	nM
K_{I2}	Threshold constant for inhibition by unphosphorylated MYB15 protein of CBF3 expression	0.3	nM
K_{I3}	Threshold constant for inhibition by ZAT12 protein of CBF3 expression	0.2	nM
v_{d1}	Maximum rate of enzymatic degradation of unphosphorylated ICE1 protein	0.1	nM/h
v_{d2}	Maximum rate of enzymatic degradation of phosphorylated ICE1 protein	0.64	nM/h
v_{d3}	Maximum rate of enzymatic degradation of CBF3 mRNA (v_{d3W} and v_{d3C} in warm and cold conditions, respectively)	0.55 (cold)/ 5.5 (warm)	nM/h
v_{d4}	Maximum rate of enzymatic degradation of CBF3 protein	1.2	nM/h
v_{d5}	Maximum rate of enzymatic degradation of phosphorylated CBF3 protein	1.8	nM/h
v_{d6}	Maximum rate of enzymatic degradation of ZAT12 mRNA	1.25	nM/h
v_{d7}	Maximum rate of enzymatic degradation of ZAT12 protein	4.125	nM/h
k_{d1}	Apparent first-order rate constant for basal degradation of ICE1 protein	0.01	h^{-1}
k_{d2}	Apparent first-order rate constant for basal degradation of phosphorylated ICE1 protein	0.01	h^{-1}
k_{d3}	Apparent first-order rate constant for basal degradation of CBF3 mRNA	0.01	h^{-1}
k_{d4}	Apparent first-order rate constant for basal degradation of CBF3 protein	0.01	h^{-1}
k_{d5}	Apparent first-order rate constant for basal degradation of phosphorylated CBF3 protein	0.01	h^{-1}
k_{d6}	Apparent first-order rate constant for basal degradation of ZAT12 mRNA	0.01	h^{-1}
k_{d7}	Apparent first-order constant for basal degradation of ZAT12 protein	0.01	h^{-1}
K_{d1}	Michaelis constant for enzymatic degradation of ICE1 protein	0.2	nM
K_{d2}	Michaelis constant for enzymatic degradation of phosphorylated ICE1 protein	0.5	nM
K_{d3}	Michaelis constant for enzymatic degradation of CBF3 mRNA	0.3	nM
K_{d4}	Michaelis constant for enzymatic degradation of CBF3 protein	0.1	nM
K_{d5}	Michaelis constant for enzymatic degradation of phosphorylated CBF3 protein	0.5	nM
K_{d6}	Michaelis constant for enzymatic degradation of ZAT12 mRNA	1.0	nM
K_{d7}	Michaelis constant for degradation of ZAT12 protein	1.25	nM

n	Hill coefficient for activation of *CBF3* expression by phosphorylated ICE1 protein	2	
m	Hill coefficient for inhibition by unphosphorylated MYB15 protein of CBF3 expression	2	
r	Hill coefficient for inhibition by ZAT12 protein of CBF3 expression	2	
s	Hill coefficient for activation of *ZAT12* expression by CBF3 protein	2	
$CRLK1_t$	Total amount of CRLK1 protein	3	nM
$CRPK1_t$	Total amount of CRPK1 protein	3	nM
$MYB15_t$	Total amount of MYB15 protein	2	nM
$MPK6_t$	Total amount of MPK6 protein	3	nM
$MPK4_t$	Total amount of MPK4 protein	3	nM
$HOS1_t$	Total amount of HOS1 protein	2	nM
$P1433_t$	Total amount of P1433 protein	2	nM

Supplementary Table 3. Sensitivity analysis for the amplitude of *CBF3* mRNA expression elicited by a cold stress, with parameters listed in the order of decreasing sensitivity.

Listed for the model for the cold response pathway governed by eqs. (4)-(17) are the 30 most sensitive parameters determined with respect to the amplitude of the peak in *CBF3* expression following a cold stress. For each of the parameters we determine the range in which the parameter value yields an amplitude of the peak between 20 and 500 times larger than the steady-state level of *CBF3* mRNA prior to the cold stress (see Supplementary Material, Section 4.2). Each parameter is varied one at a time from 10^{-3} to 10^{3} times its basal value listed in Table 2, while holding the other parameters fixed at their values given in Table 2. The parameter sensitivity is measured by determining numerically the range of values satisfying the constraint and dividing then the upper bound by the lower bound of this range. The smaller the range and the ratio, the greater the sensitivity of the system towards the parameter. For each of the 77 parameters, Figure 4A provides a graphical representation of the range satisfying the constraint.

Parameter	Definition	Basal value	Boundaries	Ratio
k_5	Rate constant for phosphorylation of MPK6 by activated CRLK1 protein	5.80	3.19-6.78	2.13
v_1	Rate of dephosphorylation of phosphorylated MPK6 protein	5.45	4.63-10.36	2.24
$\mathrm{CRLK1_t}$	Total amount of CRLK1 protein	3.00	1.32-3.60	2.73
$\mathrm{MPK6_t}$	Total amount of MPK6 protein	3.00	1.05-3.81	3.63
$\mathrm{MYB15_t}$	Total amount of MYB15 protein	2.00	1.46-5.54	3.79
K_{Ca}	Threshold for half-maximum activation of calmodulin by Ca^{2+}	0.20	0.14-0.63	4.58
k_8	Rate constant for phosphorylation of MYB15 by MPK6 protein	4.80	1.25-5.86	4.69
v_4	Rate of dephosphorylation rate of phosphorylated MYB15 protein	4.45	3.52-17.00	4.84
k_2	First-order rate constant for inactivation of activated CRLK1 protein	6.15	4.31-22.08	5.13
k_1	Apparent first-order rate constant for activation of CRLK1 by calmodulin	6.50	1.89-9.69	5.14
K_{I2}	Threshold constant for inhibition by unphosphorylated MYB15 protein of CBF3 expression	0.30	0.078-0.41	5.19
K_{a2}	Threshold constant for activation of *CBF3* expression by phosphorylated ICE1 protein	0.45	0.25-2.03	8.18
v_{d2}	Maximum rate of enzymatic degradation of phosphorylated ICE1 protein	0.64	0.20 -1.91	9.34
K_1	Michaelis constant for phosphorylation of MPK6 by activated CRLK1 protein	0.50	0.27-2.85	10.75

v_{s2}	Maximum rate of synthesis of CBF3 mRNA	18.00	1.76-25.92	14.69
v_{d3}	Maximum rate of enzymatic degradation of CBF3 mRNA	0.55(v_{d3Cold})<br>5.50(v_{d3Warm})	0.044-0.93<br>0.44-9.30	21.13
K_8	Michaelis constant for phosphorylation of MYB15 by phosphorylated MPK6 protein	0.50	0.34-6.35	21.17
K_7	Michaelis constant for dephosphorylation of phosphorylated MYB15 protein	0.50	0.014-0.90	66.67
K_{d2}	Michaelis constant for enzymatic degradation of phosphorylated ICE1 protein	0.50	0.030-3.75	127.12
K_2	Michaelis constant for dephosphorylation of phosphorylated MPK6 protein	0.50	$5.00*10^{-4}$-0.80	>1600
v_{s1}	Rate of synthesis of ICE1 protein	0.18	$1.80*10^{-4}$-0.31	>1700
v_3	Rate of transport of HOS1 out of the nucleus through putative dephosphorylation	1.95	$1.95*10^{-3}$-15.48	>7940
k_9	Rate constant for phosphorylation of ICE1 by phosphorylated MPK6 protein	15.00	1.05-$1.5*10^{4}$	>14285
v_{d1}	Maximum rate of enzymatic degradation of unphosphorylated ICE1 protein	0.10	$1.00*10^{-4}$-1.52	>15200
k_7	Rate constant for transport of cytosolic HOS1 into the nucleus through putative phosphorylation by activated CRLK1 protein	6.15	0.29-$6.15*10^{3}$	>21276
K_{I1}	Threshold constant for inhibition of ICE1 by phosphorylated MPK4 protein	0.45	0.020-450.00	>22222
v_5	Rate of dephosphorylation of phosphorylated ICE1 protein	0.80	$8.00*10^{-4}$-25.52	>31900
K_9	Michaelis constant for phosphorylation of ICE1 by phosphorylated MPK6 protein	0.45	$4.50*10^{-4}$-16.60	>36900
$HOS1_t$	Total amount of HOS1 protein	2.00	0.020-$2.00*10^{3}$	>100000

Supplementary Table 4. Parameter sensitivity analysis for the timing of the peak of *CBF3* expression elicited by a cold stress, with parameters listed in the order of decreasing sensitivity.

Listed for the model for the cold response pathway governed by eqs. (4)-(17) are the 25 most sensitive parameters determined with respect to the timing of the peak in *CBF3* expression following a cold stress. For each of the parameters we determine the range in which the parameter value yields a peak in in *CBF3* mRNA that occurs between 1h and 6 h after the cold stress (see Supplementary Material, Section 4.2). Each parameter is varied one at a time from 10^{-3} to 10^{3} times its basal value listed in Table 2, while holding the other parameters fixed at their values given in Table 2. The parameter sensitivity is measured by determining numerically the range of values satisfying the constraint and dividing then the upper bound by the lower bound of this range. The smaller the range and the ratio, the greater the sensitivity of the system towards the parameter. The ratios of parameter values extend from ~ 10 to > 10^{6}. The other non-listed parameters all have ratios > 10^{6}. For each of the 77 parameters, Figure 4B provides a graphical representation of the range satisfying the constraint.

Parameter	Definition	Basal value	Boundaries	Ratio
$\mathrm{MYB15_t}$	Total amount of MYB15 protein	2.00	1.46-15.80	10.82
v_1	Rate of dephosphorylation of phosphorylated MPK6 protein	5.45	$5.45*10^{-4}$-35.43	>6500
K_{I2}	Threshold constant for inhibition by unphosphorylated MYB15 protein of CBF3 expression	0.30	0.045-$3.00*10^{4}$	>6666
$\mathrm{MPK6_t}$	Total amount of MPK6 protein	3.00	0.45-$3.00*10^{3}$	>6666
k_1	Apparent first-order rate constant for activation of CRLK1 by calmodulin	6.50	0.078-533.00	>6833
v_{d2}	Maximum rate of enzymatic degradation of phosphorylated ICE1 protein	0.64	$6.40*10^{-4}$-4.67	>7300
k_5	Rate constant for phosphorylation of MPK6 by activated CRLK1 protein	5.80	0.75-$5.80*10^{3}$	>7692
K_{Ca}	Threshold for half-maximum activation of calmodulin by Ca^{2+}	0.20	$2.00*10^{-4}$-1.76	>8800
K_{a2}	Threshold constant for activation of *CBF3* expression by phosphorylated ICE1 protein	0.45	$4.50*10^{-4}$-4.14	>9200
v_{d3}	Maximum rate of enzymatic degradation of CBF3 mRNA	0.55(v_{d3Cold})<br>5.50(v_{d3Warm})	0.028-550.00<br>0.28-5500.00	>20000
v_{s2}	Maximum rate of synthesis of CBF3 mRNA	18.00	0.54-$1.80*10^{4}$	>33333
K_{d2}	Michaelis constant for enzymatic degradation of phosphorylated ICE1 protein	0.50	0.015-500	>33333
$\mathrm{CRLK1_t}$	Total amount of CRLK1 protein	3.00	0.090-$3.00*10^{3}$	>33333

K_{d3}	Michaelis constant for enzymatic degradation of CBF3 mRNA	0.30	$3.00*10^{-4}$-12.30	>41000
K_1	Michaelis constant for phosphorylation of MPK6 by activated CRLK1 protein	0.50	$5.00*10^{-4}$-24.00	>48000
K_7	Michaelis constant for dephosphorylation of phosphorylated MYB15 protein	0.50	$9.50*10^{-3}$-$5.00*10^{2}$	>52631
k_9	Rate constant for phosphorylation of ICE1 by phosphorylated MPK6 protein	15.00	0.26-$1.50*10^{4}$	>58823
v_{d1}	Maximum rate of enzymatic degradation of unphosphorylated ICE1 protein	0.10	$1.00*10^{-4}$-9.20	>92000
K_{I1}	Threshold constant for inhibition of ICE1 by phosphorylated MPK4 protein	0.45	$4.50*10^{-3}$-$4.50*10^{2}$	>100000
K_9	Michaelis constant for phosphorylation of ICE1 by phosphorylated MPK6 protein	0.45	$4.50*10^{-4}$-60.75	>135000
v_5	Rate of dephosphorylation of phosphorylated ICE1 protein	0.80	$8.00*10^{-4}$-112.80	>141000
K_2	Michaelis constant for dephosphorylation of phosphorylated MPK6 protein	0.50	$1.00*10^{-3}$-500.00	>500000
k_{d2}	Apparent first-order rate constant for basal degradation of phosphorylated ICE1 protein	0.010	$1.00*10^{-5}$-5.87	>587000
k_2	First-order rate constant for inactivation of activated CRLK1 protein	6.15	$6.15*10^{-3}$-$6.15*10^{3}$	>1000000

Supplementary Table 5. Parameter sensitivity analysis for the half-width of the peak of *CBF3* expression elicited by a cold stress, with parameters listed in the order of decreasing sensitivity.

Listed for the model for the cold response pathway governed by eqs. (4)-(17) are the 30 most sensitive parameters determined with respect to the half-width of the peak in *CBF3* expression following a cold stress. For each of the parameters we determine the range in which the parameter value yields a cold-induced peak in in *CBF3* mRNA with a half-width comprised between 3h and 6h. The half-width is the difference between the times at which *CBF3* mRNA after a cold stress rises up to 50% and subsequently decreases down to 50% of its maximum (see Supplementary Material, Section 4.2). Each parameter is varied one at a time from 10^{-3} to 10^{3} times its basal value listed in Table 2, while holding the other parameters fixed at their values given in Table 2. The parameter sensitivity is measured by determining numerically the range of values satisfying the constraint and dividing then the upper bound by the lower bound of this range. The smaller the range and the ratio, the greater the sensitivity of the system towards the parameter. The ratios range from < 10 to > 10^4. The other non-listed parameters in Supplementary Table 2 all have ratios > 10^4. For each of the 77 parameters, Figure 4C provides a graphical representation of the range satisfying the constraint.

Parameter	Definition	Basal value	Boundaries	Ratio
$\mathrm{CRLK1_t}$	Total amount of CRLK1 protein	3.00	2.40-3.63	1.51
k_8	Rate constant for phosphorylation of MYB15 by MPK6 protein	4.80	3.50-5.90	1.68
$\mathrm{MPK6_t}$	Total amount of MPK6 protein	3.00	2.16-3.81	1.76
v_4	Rate of dephosphorylation rate of phosphorylated MYB15 protein	4.45	3.47-6.36	1.83
k_2	First-order rate constant for inactivation of activated CRLK1 protein	6.15	4.30-8.61	2.00
K_{Ca}	Threshold for half-maximum activation of calmodulin by Ca^{2+}	0.20	0.15-0.30	2.00
k_1	Apparent first-order rate constant for activation of CRLK1 by calmodulin	6.50	4.75-9.69	2.04
K_{I2}	Threshold constant for inhibition by unphosphorylated MYB15 protein of CBF3 expression	0.30	0.19-0.41	2.05
v_{d3}	Maximum rate of enzymatic degradation of CBF3 mRNA	0.55(v_{d3Cold})<br>5.50(v_{d3Warm})	0.41-0.88<br>4.10-8.80	2.16
v_{s2}	Maximum rate of synthesis of CBF3 mRNA	18.00	9.72-26.28	2.70
K_{a2}	Threshold constant for activation of *CBF3* expression by phosphorylated ICE1 protein	0.45	0.25-0.73	2.91
K_8	Michaelis constant for phosphorylation of MYB15 by phosphorylated MPK6 protein	0.50	0.33-1.10	3.33
v_{d2}	Maximum rate of enzymatic degradation of phosphorylated ICE1 protein	0.64	0.29-1.00	3.41

v_{s1}	Rate of synthesis of ICE1 protein	0.18	0.089-0.31	3.44
K_1	Michaelis constant for phosphorylation of MPK6 by activated CRLK1 protein	0.50	0.26-0.98	3.75
K_2	Michaelis constant for dephosphorylation of phosphorylated MPK6 protein	0.50	0.22-0.85	3.93
k_5	Rate constant for phosphorylation of MPK6 by activated CRLK1 protein	5.80	0.52-6.79	13.00
K_{d2}	Michaelis constant for enzymatic degradation of phosphorylated ICE1 protein	0.50	0.20-2.60	13.00
v_1	Rate of dephosphorylation of phosphorylated MPK6 protein	5.45	4.74-69.22	14.60
K_{a1}	Michaelis constant for activation by nuclear HOS1 of phosphorylated ICE1 protein degradation	0.50	0.045-3.34	74.22
K_7	Michaelis constant for dephosphorylation of phosphorylated MYB15 protein	0.50	$5.00*10^{-4}$-0.95	>1890
v_{d1}	Maximum rate of enzymatic degradation of unphosphorylated ICE1 protein	0.10	$1.00*10^{-4}$-0.36	>3630
K_{d3}	Michaelis constant for enzymatic degradation of CBF3 mRNA	0.30	$3.00*10^{-4}$-1.23	>4100
k_9	Rate constant for phosphorylation of ICE1 by phosphorylated MPK6 protein	15.00	3.30-$1.50*10^{4}$	>4545
v_3	Rate of transport of HOS1 out of the nucleus through putative dephosphorylation	1.95	$1.95*10^{-3}$-10.34	>5300
k_7	Rate constant for transport of cytosolic HOS1 into the nucleus through putative phosphorylation by activated CRLK1 protein	6.15	0.98-$6.15*10^{3}$	>6250
K_9	Michaelis constant for phosphorylation of ICE1 by phosphorylated MPK6 protein	0.45	$4.50*10^{-4}$-3.74	>8320
v_5	Rate of dephosphorylation of phosphorylated ICE1 protein	0.80	$8.00*10^{-4}$-7.24	>9050
$HOS1_t$	Total amount of HOS1 protein	2.00	0.20-$2.00*10^{3}$	>10000
k_{d3}	Apparent first-order rate constant for basal degradation of CBF3 mRNA	0.01	$1.00*10^{-5}$-0.16	>16000

6. Replotting data from experimental figures

To compare the predictions of the computational model with experimental observations it is useful to redraw results from experimental figures where they take the form of pictures showing mRNA expression patterns in gels. To this end we follow the following steps in order to extract the experimental data by means of the software *ImageJ* for Image Processing and Analysis (https://imagej.nih.gov/ij/):

1) Download the experimental images from publications and save them as picture files.
2) Convert the image data into a grayscale or black and white array. We count the number of pixels occupied by black or grey beyond one value.
3) In ImageJ, remove some useless grey points below one value to make the picture look cleaner. Some of pixels represent disturbance or errors.
4) Select the regions of gels of mRNAs, count the number of pixels or calculate the area of bands.
5) To determine the amplitude of experimental dots, we can find a scale in experimental figures by comparing peak values between different treatment groups or choose an adequate scale arbitrary.

When converting the picture into an array, it is necessary to control the field value reasonably, avoiding values which are either too large or too small. The noise depends on the resolution of the pictures. After the background noise is eliminated, *ImageJ* automatically counts the number of selected pixels or areas of selected zones.

7. Computer codes used for numerical simulations

7.1 Response to single Ca^{2+} pulse

```
# Cold response
dCa/dt=Iin0+Iin-Iex0*Ca
dCRLK1a/dt=k1*ca^n0/(Kca^n0+ca^n0)*(CRLK1t-CRLK1a)-k2*CRLK1a
dCRPK1a/dt=k3*ca^n0/(Kca^n0+ca^n0)*(CRPK1t-CRPK1a)-k4*CRPK1a
dMPK6p/dt=k5*(MPK6t-MPK6p)*CRLK1a/(KK1+(MPK6t-MPK6p))-
v1*MPK6p/(KK2+MPK6p)
dMPK4p/dt=k6*CRLK1a*(MPK4t-MPK4p)/(KK3+MPK4t-MPK4p)-
v2*MPK4p/(KK4+MPK4p)
dHOS1n/dt=k7*CRLK1a*(HOS1t-HOS1n)/(KK5+HOS1t-HOS1n)-
v3*HOS1n/(KK6+HOS1n)
dMYB15/dt= v4*(MYB15t-MYB15)/(KK7+ MYB15t-MYB15)-k8*MPK6p* MYB15/(KK8+
MYB15)
dICE1/dt=vs1-
k9*MPK6p*ICE1/(KK9+ICE1)*KI1/(KI1+MPK4p)+v5*ICE1p/(KK10+ICE1p)-
vd1*ICE1/(KKd1+ICE1)-kd1*ICE1
dICE1p/dt=k9*MPK6p*ICE1/(KK9+ICE1)*KI1/(KI1+MPK4p)-v5*ICE1p/(KK10+ICE1p)-
vd2*ICE1p/(KKd2+ICE1p)*(HOS1n/(Ka1+HOS1n))-kd2*ICE1p
dPK1433p/dt=k10*CRPK1a*(PK1433t-PK1433p)/(KK11+PK1433t-PK1433p)-
v6*PK1433p/(KK12+PK1433p)
dCBF3m/dt=vs2*ICE1p^n/(Ka2^n+ICE1p^n)*KI2^m/(KI2^m+MYB15^m)*KI3^r/(KI3^r+Z
AT12^r)-vd3*CBF3m/(KKd3+CBF3m)-kd3*CBF3m
dCBF3/dt=ks1*CBF3m-k11*PK1433p*CBF3/(KK13+CBF3)+v7*CBF3p/(KK14+CBF3p)-
vd4*CBF3/(KKd4+CBF3)-kd4*CBF3
dCBF3p/dt=k11*PK1433p*CBF3/(KK13+CBF3)-v7*CBF3p/(KK14+CBF3p)-
vd5*CBF3p/(KKd5+CBF3p)-kd5*CBF3p
dZAT12m/dt=vs3*CBF3^s/(Ka3^s+CBF3^s)-vd6*ZAT12m/(KKd6+ZAT12m)-kd6*ZAT12m
dZAT12/dt=ks2*ZAT12m-vd7*ZAT12/(KKd7+ZAT12)-kd7*ZAT12
init CRLK1a=0.1756
init CRPK1a=0.1779
init MPK6p=0.0948
init MYB15=0.0443
init MPK4P=0.0964
init HOS1n=0.3679
init ICE1=0.2259
init ICE1p=0.4006
init PK1433p=0.2429
init CBF3m=0.1981
init CBF3=0.0750
init CBF3p=0.0059
init ZAT12m=0.0088
init ZAT12=0.0025
```

```
init Ca=0.1
param Tc=2, Td=0.05
param Iin0=0.28,Iex0= 2.8,Iin1=20
Iin=heav(t-Tc)*heav(Tc+Td-t)*Iin1
vd3=heav(t-Tc)*vd3Cold+heav(Tc-t)*vd3Warm
param vd3Cold=0.55
param vd3Warm=5.5
param n0=4,Kca=0.2
param k1=6.5,k2=6.15,k3=4.5,k4=4.2,k5=5.8,k6=4.8
param k7=6.15, k8=4.8,k9=15, k10=2.2,k11=3
param v1=5.45,v2=4.45,v3=1.95,v4=4.45,v5=0.8,v6=1.05,v7=1
param KK1=0.5,KK2=0.5,KK3=0.5,KK4=0.5,KK5=0.5,KK6=0.5,KK7=0.5
param KK8=0.5,KK9=0.45,KK10=0.8,KK11=0.65,KK12=0.65,KK13=0.6,KK14=0.58
param vs1=0.18,vs2=18,vs3=4.75,ks1=0.25,ks2=0.5
param Ka1=0.5,Ka2=0.45,Ka3=0.1,KI1=0.45,KI2=0.3,KI3=0.5
param vd1=0.1,vd2=0.64, vd4=1.2,vd5=1.8,vd6=1.25, vd7=4.125
param kd1=0.01,kd2=0.01,kd3=0.01,kd4=0.01,kd5=0.01,kd6=0.01,kd7=0.01
param KKd1=0.2,KKd2=0.5,KKd3=0.3,KKd4=0.1,KKd5=0.5,KKd6=1.0,KKd7=1.25
param  n=2,m=2,r=2,s=2
param CRLK1t=3,CRPK1t=3,MPK6t=3, MPK4t=3, HOS1t=2, MYB15t=2, PK1433t=2
@ total=30,dt=0.01,MAXSTOR=100000,bounds=10000
@ nplot=2,yp1=CBF3M,yp2=ca
done
```

7.2 Response to Ca^{2+} oscillations

```
# Cold response with calcium oscillation
dCRLK1a/dt=k1*ca^n0/(Kca^n0+ca^n0)*(CRLK1t-CRLK1a)-k2*CRLK1a
dCRPK1a/dt=k3*ca^n0/(Kca^n0+ca^n0)*(CRPK1t-CRPK1a)-k4*CRPK1a
dMPK6p/dt=k5*(MPK6t-MPK6p)*CRLK1a/(KK1+(MPK6t-MPK6p))-
v1*MPK6p/(KK2+MPK6p)
dMPK4p/dt=k6*CRLK1a*(MPK4t-MPK4p)/(KK3+MPK4t-MPK4p)-
v2*MPK4p/(KK4+MPK4p)
dHOS1n/dt=k7*CRLK1a*(HOS1t-HOS1n)/(KK5+HOS1t-HOS1n)-
v3*HOS1n/(KK6+HOS1n)
dMYB15/dt= v4*(MYB15t-MYB15)/(KK7+ MYB15t-MYB15)-k8*MPK6p* MYB15/(KK8+
MYB15)
dICE1/dt=vs1-
k9*MPK6p*ICE1/(KK9+ICE1)*KI1/(KI1+MPK4p)+v5*ICE1p/(KK10+ICE1p)-
vd1*ICE1/(KKd1+ICE1)-kd1*ICE1
dICE1p/dt=k9*MPK6p*ICE1/(KK9+ICE1)*KI1/(KI1+MPK4p)-v5*ICE1p/(KK10+ICE1p)-
vd2*ICE1p/(KKd2+ICE1p)*(HOS1n/(Ka1+HOS1n))-kd2*ICE1p
dPK1433p/dt=k10*CRPK1a*(PK1433t-PK1433p)/(KK11+PK1433t-PK1433p)-
v6*PK1433p/(KK12+PK1433p)
```

```
dCBF3m/dt=vs2*ICE1p^n/(Ka2^n+ICE1p^n)*KI2^m/(KI2^m+MYB15^m)*KI3^r/(KI3^r+Z
AT12^r)-vd3*CBF3m/(KKd3+CBF3m)-kd3*CBF3m
dCBF3/dt=ks1*CBF3m-k11*PK1433p*CBF3/(KK13+CBF3)+v7*CBF3p/(KK14+CBF3p)-
vd4*CBF3/(KKd4+CBF3)-kd4*CBF3
dCBF3p/dt=k11*PK1433p*CBF3/(KK13+CBF3)-v7*CBF3p/(KK14+CBF3p)-
vd5*CBF3p/(KKd5+CBF3p)-kd5*CBF3p
dZAT12m/dt=vs3*CBF3^s/(Ka3^s+CBF3^s)-vd6*ZAT12m/(KKd6+ZAT12m)-kd6*ZAT12m
dZAT12/dt=ks2*ZAT12m-vd7*ZAT12/(KKd7+ZAT12)-kd7*ZAT12

dCa/dt=cav0+cav1*beta-cav2(Ca)+cav3(Ca,y)+cakf*Y-cak*Ca
dY/dt=cav2(Ca)-cav3(Ca,y)-cakf*Y

init CRLK1a=0.1756
init CRPK1a=0.1779
init MPK6p=0.0948
init MYB15=0.0443
init MPK4P=0.0964
init HOS1n=0.3679
init ICE1=0.2259
init ICE1p=0.4006
init PK1433p=0.2429
init CBF3m=0.1981
init CBF3=0.0750
init CBF3p=0.0059
init ZAT12m=0.0088
init ZAT12=0.0025
init Ca=0.1
init y=0.6
param betaM=0.9,betaH=2.1,Tc=2
beta=(betaM)*exp(-betaH*(t-Tc))*heav(t-Tc)
param cav0=25,cav1=182.5,cavM2=1625,cavM3=12500
param caKR=2,caKA=0.9,caK2=1,cakf=25,cak=250
param can=2,cam=2,cap=4
cav2(ca)=cavM2*Ca^can/(caK2^can+Ca^can)
cav3(ca,y)=cavM3*Y^cam/(caKR^cam+Y^cam)*Ca^cap/(caKA^cap+Ca^cap)
vd3=heav(t-Tc)*vd3Cold+heav(Tc-t)*vd3Warm
param vd3Cold=0.55
param vd3Warm=5.5
param n0=4,Kca=0.2
param k1=6.5,k2=6.15,k3=4.5,k4=4.2,k5=5.8,k6=4.8
param k7=6.15, k8=4.8,k9=15, k10=2.2,k11=3
param v1=5.45,v2=4.45,v3=1.95,v4=4.45,v5=0.8,v6=1.05,v7=1
param KK1=0.5,KK2=0.5,KK3=0.5,KK4=0.5,KK5=0.5,KK6=0.5,KK7=0.5
param KK8=0.5,KK9=0.45,KK10=0.8,KK11=0.65,KK12=0.65,KK13=0.6,KK14=0.58
param vs1=0.18,vs2=18,vs3=4.75,ks1=0.25,ks2=0.5
param Ka1=0.5,Ka2=0.45,Ka3=0.1,KI1=0.45,KI2=0.3,KI3=0.5
```

```
param vd1=0.1,vd2=0.64, vd4=1.2,vd5=1.8,vd6=1.25, vd7=4.125
param kd1=0.01,kd2=0.01,kd3=0.01,kd4=0.01,kd5=0.01,kd6=0.01,kd7=0.01
param KKd1=0.2,KKd2=0.5,KKd3=0.3,KKd4=0.1,KKd5=0.5,KKd6=1.0,KKd7=1.25
param  n=2,m=2,r=2,s=2
param CRLK1t=3,CRPK1t=3,MPK6t=3, MPK4t=3, HOS1t=2, MYB15t=2, PK1433t=2
@ total=30,dt=0.00001,MAXSTOR=100000,bounds=10000
@ nplot=2,yp1=CBF3M,yp2=ca
done
```

7.3 Response to successive cold stresses

```
# Cold response to successive Calcium signals
dCa/dt=Iin0+Iin-Iex0*Ca
dCRLK1a/dt=k1*ca^n0/(Kca^n0+ca^n0)*(CRLK1t-CRLK1a)-k2*CRLK1a
dCRPK1a/dt=k3*ca^n0/(Kca^n0+ca^n0)*(CRPK1t-CRPK1a)-k4*CRPK1a
dMPK6p/dt=k5*(MPK6t-MPK6p)*CRLK1a/(KK1+(MPK6t-MPK6p))-
v1*MPK6p/(KK2+MPK6p)
dMPK4p/dt=k6*CRLK1a*(MPK4t-MPK4p)/(KK3+MPK4t-MPK4p)-
v2*MPK4p/(KK4+MPK4p)
dHOS1n/dt=k7*CRLK1a*(HOS1t-HOS1n)/(KK5+HOS1t-HOS1n)-
v3*HOS1n/(KK6+HOS1n)
dMYB15/dt= v4*(MYB15t-MYB15)/(KK7+ MYB15t-MYB15)-k8*MPK6p* MYB15/(KK8+
MYB15)
dICE1/dt=vs1-
k9*MPK6p*ICE1/(KK9+ICE1)*KI1/(KI1+MPK4p)+v5*ICE1p/(KK10+ICE1p)-
vd1*ICE1/(KKd1+ICE1)-kd1*ICE1
dICE1p/dt=k9*MPK6p*ICE1/(KK9+ICE1)*KI1/(KI1+MPK4p)-v5*ICE1p/(KK10+ICE1p)-
vd2*ICE1p/(KKd2+ICE1p)*(HOS1n/(Ka1+HOS1n))-kd2*ICE1p
dPK1433p/dt=k10*CRPK1a*(PK1433t-PK1433p)/(KK11+PK1433t-PK1433p)-
v6*PK1433p/(KK12+PK1433p)
dCBF3m/dt=vs2*ICE1p^n/(Ka2^n+ICE1p^n)*KI2^m/(KI2^m+MYB15^m)*KI3^r/(KI3^r+Z
AT12^r)-vd3*CBF3m/(KKd3+CBF3m)-kd3*CBF3m
dCBF3/dt=ks1*CBF3m-k11*PK1433p*CBF3/(KK13+CBF3)+v7*CBF3p/(KK14+CBF3p)-
vd4*CBF3/(KKd4+CBF3)-kd4*CBF3
dCBF3p/dt=k11*PK1433p*CBF3/(KK13+CBF3)-v7*CBF3p/(KK14+CBF3p)-
vd5*CBF3p/(KKd5+CBF3p)-kd5*CBF3p
dZAT12m/dt=vs3*CBF3^s/(Ka3^s+CBF3^s)-vd6*ZAT12m/(KKd6+ZAT12m)-kd6*ZAT12m
dZAT12/dt=ks2*ZAT12m-vd7*ZAT12/(KKd7+ZAT12)-kd7*ZAT12

init CRLK1a=0.1756
init CRPK1a=0.1779
init MPK6p=0.0948
init MYB15=0.0443
init MPK4P=0.0964
init HOS1n=0.3679
```

```
init ICE1=0.2259
init ICE1p=0.4006
init PK1433p=0.2429
init CBF3m=0.1981
init CBF3=0.0750
init CBF3p=0.0059
init ZAT12m=0.0088
init ZAT12=0.0025
init Ca=0.1
param Tp=3, Td=0.05
Tc1=Tp/2
Tc2=Tp*3/2
Tc3=Tp*5/2
Tc4=Tp*7/2
param Iin0=0.28,Iex0= 2.8,Iin1=20
Iin=heav(t-Tc1)*heav(Tc1+Td-t)*Iin1+heav(t-Tc2)*heav(Tc2+Td-t)*Iin1+heav(t-
Tc3)*heav(Tc3+Td-t)*Iin1+heav(t-Tc4)*heav(Tc4+Td-t)*Iin1
vd3=(heav(t-Tc1)*heav(Tp-t)+heav(t-Tc2)*heav(2*Tp-t)+heav(t-Tc3)*heav(3*Tp-t)+heav(t-
Tc4)*heav(4*Tp-t))*vd3Cold+(heav(Tc1-t)+heav(Tc2-t)*heav(t-Tp)+heav(Tc3-t)*heav(t-
2*Tp)+heav(Tc4-t)*heav(t-3*Tp))*vd3Warm
param vd3Cold=0.55
param vd3Warm=5.5
param n0=4,Kca=0.2
param k1=6.5,k2=6.15,k3=4.5,k4=4.2,k5=5.8,k6=4.8
param k7=6.15, k8=4.8,k9=15, k10=2.2,k11=3
param v1=5.45,v2=4.45,v3=1.95,v4=4.45,v5=0.8,v6=1.05,v7=1
param KK1=0.5,KK2=0.5,KK3=0.5,KK4=0.5,KK5=0.5,KK6=0.5,KK7=0.5
param KK8=0.5,KK9=0.45,KK10=0.8,KK11=0.65,KK12=0.65,KK13=0.6,KK14=0.58
param vs1=0.18,vs2=18,vs3=4.75,ks1=0.25,ks2=0.5
param Ka1=0.5,Ka2=0.45,Ka3=0.1,KI1=0.45,KI2=0.3,KI3=0.5
param vd1=0.1,vd2=0.64, vd4=1.2,vd5=1.8,vd6=1.25, vd7=4.125
param kd1=0.01,kd2=0.01,kd3=0.01,kd4=0.01,kd5=0.01,kd6=0.01,kd7=0.01
param KKd1=0.2,KKd2=0.5,KKd3=0.3,KKd4=0.1,KKd5=0.5,KKd6=1.0,KKd7=1.25
param  n=2,m=2,r=2,s=2
param CRLK1t=3,CRPK1t=3,MPK6t=3, MPK4t=3, HOS1t=2, MYB15t=2, PK1433t=2
aux vd3=vd3
@ total=13,dt=0.01,MAXSTOR=100000,bounds=10000
@ nplot=2,yp1=CBF3M,yp2=ca
done
```

7.4 Circadian gating

```
# Cold response under circadian gating
dCa/dt=Iin0+Iin-Iex0*Ca
dCRLK1a/dt=k1*ca^n0/(Kca^n0+ca^n0)*(CRLK1t-CRLK1a)-k2*CRLK1a
```

```
dCRPK1a/dt=k3*ca^n0/(Kca^n0+ca^n0)*(CRPK1t-CRPK1a)-k4*CRPK1a
dMPK6p/dt=k5*(MPK6t-MPK6p)*CRLK1a/(KK1+(MPK6t-MPK6p))-
v1*MPK6p/(KK2+MPK6p)
dMPK4p/dt=k6*CRLK1a*(MPK4t-MPK4p)/(KK3+MPK4t-MPK4p)-
v2*MPK4p/(KK4+MPK4p)
dHOS1n/dt=k7*CRLK1a*(HOS1t-HOS1n)/(KK5+HOS1t-HOS1n)-
v3*HOS1n/(KK6+HOS1n)
dMYB15/dt= v4*(MYB15t-MYB15)/(KK7+ MYB15t-MYB15)-k8*MPK6p* MYB15/(KK8+
MYB15)
dICE1/dt=vs1-
k9*MPK6p*ICE1/(KK9+ICE1)*KI1/(KI1+MPK4p)+v5*ICE1p/(KK10+ICE1p)-
vd1*ICE1/(KKd1+ICE1)-kd1*ICE1
dICE1p/dt=k9*MPK6p*ICE1/(KK9+ICE1)*KI1/(KI1+MPK4p)-v5*ICE1p/(KK10+ICE1p)-
vd2*ICE1p/(KKd2+ICE1p)*(HOS1n/(Ka1+HOS1n))-kd2*ICE1p
dPK1433p/dt=k10*CRPK1a*(PK1433t-PK1433p)/(KK11+PK1433t-PK1433p)-
v6*PK1433p/(KK12+PK1433p)
dCBF3m/dt=vs2*g*ICE1p^n/(Ka2^n+ICE1p^n)*KI2^m/(KI2^m+MYB15^m)*KI3^r/(KI3^r+
ZAT12^r)-vd3*CBF3m/(KKd3+CBF3m)-kd3*CBF3m
dCBF3/dt=ks1*CBF3m-k11*PK1433p*CBF3/(KK13+CBF3)+v7*CBF3p/(KK14+CBF3p)-
vd4*CBF3/(KKd4+CBF3)-kd4*CBF3
dCBF3p/dt=k11*PK1433p*CBF3/(KK13+CBF3)-v7*CBF3p/(KK14+CBF3p)-
vd5*CBF3p/(KKd5+CBF3p)-kd5*CBF3p
dZAT12m/dt=vs3*CBF3^s/(Ka3^s+CBF3^s)-vd6*ZAT12m/(KKd6+ZAT12m)-kd6*ZAT12m
dZAT12/dt=ks2*ZAT12m-vd7*ZAT12/(KKd7+ZAT12)-kd7*ZAT12
C=C0+C1*(1/2*(1+sin(2*pi*(t-2)/pclock)))^q
g=C^h/(Kc^h+C^h)

init CRLK1a=0.1756
init CRPK1a=0.1779
init MPK6p=0.0948
init MYB15=0.0443
init MPK4P=0.0964
init HOS1n=0.3679
init ICE1=0.2259
init ICE1p=0.4006
init PK1433p=0.2429
init CBF3m=0.1981
init CBF3=0.0750
init CBF3p=0.0059
init ZAT12m=0.0088
init ZAT12=0.0025
init Ca=0.1

tt=mod(t,pcold)
Iin=heav(tt-Tc)*heav(Tc+Td-tt)*Iin1
vd3=heav(tt-Tc)*vd3Cold+heav(Tc-tt)*vd3Warm
```

```
param C0=0.01,C1=1,phi=6
param q=4
param Kc=0.15
param h=4
param pclock=24
param pcold=24
param Tc=2, Td=0.05
param Iin0=0.28,Iex0= 2.8,Iin1=20
param vd3Cold=0.55
param vd3Warm=5.5
param n0=4,Kca=0.2
param k1=6.5,k2=6.15,k3=4.5,k4=4.2,k5=5.8,k6=4.8
param k7=6.15, k8=4.8,k9=15, k10=2.2,k11=3
param v1=5.45,v2=4.45,v3=1.95,v4=4.45,v5=0.8,v6=1.05,v7=1
param KK1=0.5,KK2=0.5,KK3=0.5,KK4=0.5,KK5=0.5,KK6=0.5,KK7=0.5
param KK8=0.5,KK9=0.45,KK10=0.8,KK11=0.65,KK12=0.65,KK13=0.6,KK14=0.58
param vs1=0.18,vs2=18,vs3=4.75,ks1=0.25,ks2=0.5
param Ka1=0.5,Ka2=0.45,Ka3=0.1,KI1=0.45,KI2=0.3,KI3=0.5
param vd1=0.1,vd2=0.64, vd4=1.2,vd5=1.8,vd6=1.25, vd7=4.125
param kd1=0.01,kd2=0.01,kd3=0.01,kd4=0.01,kd5=0.01,kd6=0.01,kd7=0.01
param KKd1=0.2,KKd2=0.5,KKd3=0.3,KKd4=0.1,KKd5=0.5,KKd6=1.0,KKd7=1.25
param  n=2,m=2,r=2,s=2
param CRLK1t=3,CRPK1t=3,MPK6t=3, MPK4t=3, HOS1t=2, MYB15t=2, PK1433t=2
aux Iin=Iin
aux vd3=vd3
aux clock=C
@ S1=Tc, SLO1=0, SHI1=24
@ total=96,dt=0.01,MAXSTOR=100000,bounds=1000
@ nplot=4, yp1=Ca, yp2=CBF3m, yp3=CBF3, yp4=clock
done
```

* * * * *

8. References cited in Supplementary Material

Agarwal, M., Hao, Y., Kapoor, A., Dong, C., Fujii, H., Zheng, X., et al. (2006). A R2R3 type MYB transcription factor is involved in the cold regulation of CBF genes and in acquired freezing tolerance. J. Biol. Chem. 281, 37636–37645. doi: 10.1074/jbc.M605895200

Allen, G. J., Chu, S. P., Harrington, C. L., Schumacher, K., Hoffmann, T., Tang, Y. Y., et al. (2001). A defined range of guard cell calcium oscillation parameters encodes stomatal movements. Nature. 411, 1053-1057. doi: 10.1038/35082575

Chinnusamy, V., Ohta, M., Kanrar, S., Lee, B., Hong, X., Agarwal, M., and Zhu, J. K. (2003). ICE1: A regulator of cold-induced transcriptome and freezing tolerance in *Arabidopsis*. Genes Dev. 17, 1043–1054. doi: 10.1101/gad.1077503

Dong, M. A., Farré, E. M., and Thomashow, M. F. (2011). CIRCADIAN CLOCK-ASSOCIATED 1 and LATE ELONGATED HYPOCOTYL regulate expression of the C-REPEAT BINDING FACTOR (CBF) pathway in *Arabidopsis*. Proc. Natl. Acad. Sci. USA. 108, 7241-7246. doi: 10.1073/pnas.1103741108

Dupont, G., Berridge, M. J., and Goldbeter, A. (1991). Signal-induced Ca^{2+} oscillations: properties of a model based on Ca^{2+}-induced Ca^{2+} release. Cell Calcium. 12, 73-85. doi: 10.1016/0143-4160(91)90010-c

Goldbeter, A., Dupont, G., and Berridge, M. J. (1990). Minimal model for signal-induced Ca^{2+} oscillations and for their frequency encoding through protein phosphorylation. Proc. Natl. Acad. Sci. USA. 87, 1461-1465. doi: 10.1073/pnas.87.4.1461

Medina, J., Catalá, R., and Salinas, J. (2011) The CBFs: three arabidopsis transcription factors to cold acclimate. Plant Science. 180, 3-11. doi: 10.1016/j.plantsci.2010.06.019

Plieth, C. (1999). Temperature sensing by plants: calcium-permeable channels as primary sensors–a model. J. Membr. Biol. 172, 121-127. doi: 10.1007/s002329900590

Vogel, J. T., Zarka D. G., Van Buskirk, H. A., Fowler S. G., and Thomashow, M. F. (2005). Roles of the CBF2 and ZAT12 transcription factors in configuring the low temperature transcriptome of *Arabidopsis*. Plant J. 41, 195–211. doi: 10.1111/j.1365-313X.2004.02288.x

Zarka, D. G., Vogel, J. T., Cook, D., and Thomashow, M. F. (2003). Cold induction of *Arabidopsis* CBF genes involves multiple ICE (Inducer of CBF Expression) promoter elements and a cold-regulatory circuit that is desensitized by low temperature. Plant Physiol. 133, 910-918. doi: 10.1104/pp.103.027169is
